# Integrating Hyperspectral Reflectance-Based Phenotyping and SSR Marker-Based Genotyping for Assessing the Salt Tolerance of Wheat Genotypes under Real Field Conditions

**DOI:** 10.3390/plants13182610

**Published:** 2024-09-19

**Authors:** Salah El-Hendawy, Muhammad Bilawal Junaid, Nasser Al-Suhaibani, Ibrahim Al-Ashkar, Abdullah Al-Doss

**Affiliations:** Department of Plant Production, College of Food and Agriculture Sciences, King Saud University, KSA, P.O. Box 2460, Riyadh 11451, Saudi Arabia

**Keywords:** high-throughput phenotyping, Mantel test, morpho-physiological traits, plant breeding, recombinant inbred lines, vegetation indices, water indices

## Abstract

Wheat breeding programs are currently focusing on using non-destructive and cost-effective hyperspectral sensing tools to expeditiously and accurately phenotype large collections of genotypes. This approach is expected to accelerate the development of the abiotic stress tolerance of genotypes in breeding programs. This study aimed to assess salt tolerance in wheat genotypes using non-destructive canopy spectral reflectance measurements as an alternative to direct laborious and time-consuming phenological selection criteria. Eight wheat genotypes and sixteen F_8_ RILs were tested under 150 mM NaCl in real field conditions for two years. Fourteen spectral reflectance indices (SRIs) were calculated from the spectral data, including vegetation SRIs and water SRIs. The effectiveness of these indices in assessing salt tolerance was compared with four morpho-physiological traits using genetic parameters, SSR markers, the Mantel test, hierarchical clustering heatmaps, stepwise multiple linear regression, and principal component analysis (PCA). The results showed significant differences (*p* ≤ 0.001) among RILs/cultivars for both traits and SRIs. The heritability, genetic gain, and genotypic and phenotypic coefficients of variability for most SRIs were comparable to those of measured traits. The SRIs effectively differentiated between salt-tolerant and sensitive genotypes and exhibited strong correlations with SSR markers (R^2^ = 0.56–0.89), similar to the measured traits and allelic data of 34 SSRs. A strong correlation (r = 0.27, *p* < 0.0001) was found between the similarity coefficients of SRIs and SSR data, which was higher than that between measured traits and SSR data (r = 0.20, *p* < 0.0003) based on the Mantel test. The PCA indicated that all vegetation SRIs and most water SRIs were grouped with measured traits in a positive direction and effectively identified the salt-tolerant RILs/cultivars. The PLSR models, which were based on all SRIs, accurately and robustly estimated the various morpho-physiological traits compared to using individual SRIs. The study suggests that various SRIs can be integrated with PLSR in wheat breeding programs as a cost-effective and non-destructive tool for phenotyping and screening large wheat populations for salt tolerance in a short time frame. This approach can replace the need for traditional morpho-physiological traits and accelerate the development of salt-tolerant wheat genotypes.

## 1. Introduction

Salinity is a major concern that seriously impacts food security, especially in arid and semiarid regions. The high temperatures and evaporation rates, coupled with low and inconsistent precipitation exacerbate salinity problems in these regions year by year. Moreover, the scarcity of freshwater in these regions forces farmers to resort to using brackish water for irrigation, adding another layer of complexity to the problem [1]. Shokat and Großkinsky [2] found that using brackish water for irrigation, even with a low electrical conductivity of 0.5 dS m^−1^ (5 mM or 320 ppm), can still introduce enough salt to induce salinity stress in arid conditions. This is because high evaporation rates quickly evaporate water, leaving salt to accumulate on the soil surface. Additionally, salinity currently impacts the productivity of 30% of irrigated land (76 million hectares (Mha)) and 20% of arable land (32 Mha). Approximately 12 Mha of productive lands are salinized each year. By 2050, nearly half of the world’s arable land is expected to become salinized, reducing its capacity to produce food crops [3,4,5,6]. These alarming statistics highlight the threat that salinity poses to global food security as the population continues to grow. Therefore, it is crucial that we address this issue with comprehensive, efficient, and sustainable solutions to ensure food security in various regions worldwide.

Bread wheat is a vital cereal crop cultivated globally, accounting for 30% of total grain production and providing 45% of cereal nutrition. It serves as a staple food for a third of the world’s population, supplying 20% of calories and protein in human diets. Wheat is grown on 221 Mha, producing approximately 770 million metric tons. To meet the projected demand by 2050, wheat production must increase by almost 70% [7,8,9]. However, bread wheat shows moderate salt tolerance, with yield declining at 60–80 mM NaCl, dropping to 50% at 130 mM NaCl, and being completely lost at 200 mM NaCl [10,11]. Therefore, enhancing the salt tolerance of wheat genotypes is crucial for sustaining bread wheat production in saline soils or when irrigated with saline water. Research by Zada et al. [12] showed that improving stress tolerance in wheat genotypes could increase yield by 25%. Interestingly, introducing salt-tolerant wheat genotypes to farmers is a cost-effective long-term solution to boost wheat production in saline regions, outperforming traditional agronomic practices like leaching and gypsum application. However, enhancing salt tolerance in genotypes is a challenging task due to limited genetic diversity, infrequent use of multiple traits for evaluating salt tolerance, difficulties with in-depth multidimensional descriptions of different plant traits using conventional methods, and a lack of experiments that assess salt tolerance for a range of genotypes in real field conditions [6,13,14,15,16]

While there are numerous morpho-physiological plant traits that can effectively serve as screening criteria to assess salt tolerance in genotypes, plant breeders are often hesitant to utilize them. This reluctance stems from the time-consuming, destructive, and costly nature of assessing these traits using traditional methods and plant sampling. For example, in a previous study, we found that plant dry weight (PDW), pigment contents, and leaf relative water content (LRWC) are effective screening criteria for distinguishing between salt-tolerant and sensitive genotypes in real field conditions at both phenotypic and genotypic levels [17]. However, the traditional methods and instruments used to assess these traits, as described in the Materials and Methods Section of this paper, are time-consuming, labor-intensive, destructive, and expensive. This is especially true when these traits need to be assessed at different growth stages for a large number of genotypes, a common requirement in genomics-assisted plant breeding. To address these challenges and harness genomics advancements for the rapid development of new genotypes tolerant to abiotic stress, it is essential to employ high-throughput phenotyping techniques (HTPTs). These tools facilitate the quick and efficient collection of high-quality phenotypic data on various plant traits associated with abiotic stress in a cost-effective and non-invasive manner. They allow continuous growth monitoring without the need for destructive analysis and expand the range of genotypes that can be tested in the study [18,19,20,21,22,23,24]. The combination of genomics and HTPT enables the quick screening of multiple plant lines to pinpoint desired traits, leading to more efficient and targeted breeding efforts. This integration also helps in deciphering the genetic factors behind plant traits linked to salinity stress, providing breeders with valuable information to make informed decisions. Ultimately, this synergy has the potential to propel wheat breeding initiatives forward and bolster food security amidst the challenges posed by climate change.

Proximal remote sensing, like hyperspectral spectroradiometers, is one of the HTPTs that allows for a quick and non-invasive assessment of multiple plant traits across a large number of genotypes. The sensors of this tool can detect stress-related changes in the canopy’s physical and chemical properties by analyzing specific spectral regions in the visible (VIS; 400–700 nm), near-infrared (NIR; 700–1300 nm), and shortwave infrared (SWIR; 1300–2500 nm) electromagnetic spectrum [1,25,26]. Salinity stress affects various aspects of plant growth and physiology, such as photosynthetic pigments, biomass accumulation, leaf structure, leaf area index, biochemical components, and water content. These changes in the canopy’s characteristics alter its spectral signatures in the VIS to SWIR domains. This relationship allows for the use of spectral reflectance data in plant phenomics to assess stress-induced plant traits accurately and non-invasively. For example, changes in leaf pigments and photosynthetic function impact the spectral reflectance at 680 and 740 nm wavelengths. Modifications in the internal structure of leaves, LAI, and aboveground biomass influence the spectral reflectance at different wavelengths in the NIR spectrum. Reductions in LRWC alter the spectral reflectance at multiple bands around 970, 1100, 1200, 1400, 1900, 2100, and 2250 nm [1,24,27,28,29,30]. However, to accurately and easily assess plant traits using spectral reflectance data, various spectral reflectance indices (SRIs) have been derived from these data through simple mathematical equations. These equations include simple ratios, normalization, differences, standard differences, and derivatives. Fortunately, various SRIs have been successfully used as proxies to estimate different plant traits, particularly under a range of biotic and abiotic stresses. For example, the normalized difference vegetation index (NDVI) and the simple ratio (SR) are common indices used to assess plant traits related to pigment contents, photosynthetic efficiency, biomass accumulation, and yield in different crops under normal and stress conditions. The NDVI is calculated as (R_NIR_ − R_Red_)/(R_NIR_ + R_Red_), while the SR is calculated as R_NIR_/R_Red_ [23,30,31,32]. In their study, Colovic et al. [30] identified the following red-edge indices as the most effective predictors of maize physiological traits: normalized difference red-edge (R_790_ − R_770_)/(R_790_ + R_770_), double difference index (R_749_ − R_726_)/(R_701_ − R_672_), and chlorophyll red-edge (R_850_/R_730_) − 1. Several water SRIs were derived from spectral data, including the simple ratio water index (RWI = (R_970_/R_900_)), various normalized water indices (NWIs) that target minor water absorption bands in the NIR spectrum (970 nm), and normalized difference moisture index (NDMI = (R_2200_ − R_1100_)/(R_2200_ + R_1100_)), which are commonly used to track plant water status and stress levels [27,30,33,34,35]. Furthermore, Rud et al. [36] developed seven vegetation SRIs using the blue (420–470 nm), green (510–580 nm), red (660–680 nm), and NIR (700–800 nm) spectrum regions to assess the salt tolerance of eggplant grown under varying salinity levels. Zhang et al. [37] found that spectral reflectance indices (SRIs) incorporating the near-infrared (NIR) band at 1321 nm and the shortwave infrared (SWIR) band at 2264 nm were effective in detecting the response of cotton growth to salt stress. Consequently, plant breeders can use various SRIs as indirect selection tools to evaluate and enhance genotypes for their tolerance to abiotic stress. This approach can replace costly and damaging traditional direct phenological selection criteria, enabling breeders to quickly and non-invasively evaluate a large number of genotypes. Ultimately, this approach can potentially enhance the efficiency of breeding programs for improving stress-tolerant genotypes if the SRI tools are integrated with molecular markers.

Molecular markers are versatile tools that can be used to compare genotypes in different environmental conditions. They are not restricted by crop growth stages and stress levels, allowing for the integration of multiple tolerance traits into a single effective genotype. Furthermore, they offer objective data for analysis. Simple sequence repeat (SSR) or microsatellite markers are valuable tools for studying genetic variation in wheat. They are commonly found in wheat genomes, easy to identify, have specific chromosomal locations, exhibit co-dominant inheritance, are cost-effective, high-throughput, multi-allelic, unbiased, repeatable, and display high rates of polymorphism [14,38,39,40]. SSR markers are highly polymorphic and have multiple alleles, making them ideal for analyzing genotype relationships with fewer markers [41]. Recent studies have emphasized the significance of SSR markers in assessing salt tolerance in wheat genotypes and identifying the most salt-tolerant varieties by associating them with specific phenotypic traits. Several successful SSR primers, including Xwmc170, Gwm 312, and Xgwm312, have been used to assess the salt tolerance of wheat genotypes. These primers are effective in detecting the Nax1 gene, which is a crucial marker for Na^+^ exclusion [42,43,44]. Therefore, a SSR analysis of DNA polymorphism is essential for pinpointing key genes associated with salt tolerance in wheat.

Enhancing salt tolerance in new wheat genotypes and ensuring their genetic potential is a key objective for breeders. They usually use traditional phenotypic traits as screening criteria and SSR markers to assess the genetic diversity of candidate genotypes. To our knowledge, no studies have used SRIs from high-throughput phenotypic data instead of expensive and harmful direct phenological selection criteria, along with SSR markers to assess salt tolerance in wheat genotypes or other field crops under real field conditions. To bridge this gap, we utilized a combination of seven vegetation SRIs, seven water SRIs, four morpho-physiological traits, and six SSR markers to assess the genetic diversity of 16 F_8_ recombinant inbred lines (RILs) and eight wheat genotypes grown under high salinity levels in real field conditions. Therefore, this study was conducted with the following objectives: (1) compare and assess the salt tolerance of different wheat genotypes using traditional morpho-physiological traits, different SRIs, and genotypic data from SSR markers; (2) compare the effectiveness of SRIs and morpho-physiological traits as screening criteria for assessing salt tolerance among genotypes; (3) validate the SRIs as screening criteria by analyzing their correlation with SSR markers; and (4) validate the SRIs as high-throughput phenotyping tools for the rapid and cost-effective estimation of morpho-physiological traits, typically measured in a destructive and costly manner. The findings of this study will allow for the rapid and cost-effective improvement of salt tolerance in wheat genotypes in breeding programs.

## 2. Results

### 2.1. Analysis of Variance

The ANOVA results for mean square values showed highly significant differences (*p* ≥ 0.001) among genotypes for all destructively measured morpho-physiological traits and SRIs in each season and combined two seasons (Table 1). The leaf relative water content (LRWC) and total chlorophyll content (Chlt) showed highly significant differences (*p* ≥ 0.001), while all SRIs, except for the dry matter content index (DMCI), exhibited significant differences (*p* ≥ 0.05, 0.01, and 0.001) among seasons. The interaction between genotype and season was highly significant for LRWC and Chlt (*p* ≥ 0.001), as well as for all SRIs (*p* ≥ 0.05, 0.01, and 0.001) (Table 1).

### 2.2. Genotypic Performance in Destructively Measured Morpho-Physiological Traits and Spectral Reflectance Indices

**Figure 1:**
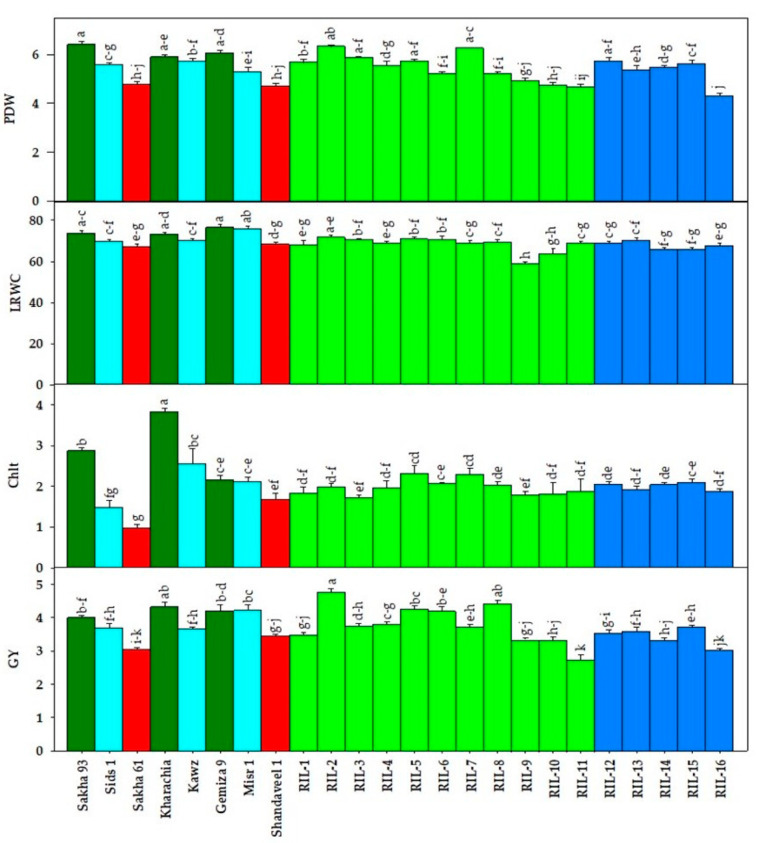
Genotypic variability in four destructively measured traits among 24 RILs/cultivars under salinity conditions. PDW, LRWC, Chlt, and GY indicate plant dry weight (g plant^−1^), leaf relative water content (%), total chlorophyll content (mg g^−1^ FW), and grain yield (ton ha^−1^), respectively. Bars with the same letters are not significantly different at the 0.05 level based on Tukey’s test. Data for each trait are the average of two seasons and three replicates, with bars representing the standard error (n = 6). The dark green color represents salt-tolerant genotypes; the red color represents salt-sensitive genotypes; the aqua color indicates moderately tolerant genotypes; the light green color represents 11 RILs resulting from the cross between salt-tolerant (Sakha 93) and salt-sensitive (Sakha 61) genotypes; and the blue color represents 5 RILs resulting from the cross between salt-tolerant and moderately salt-tolerant (Sids 1) genotypes.

**Figure 2:**
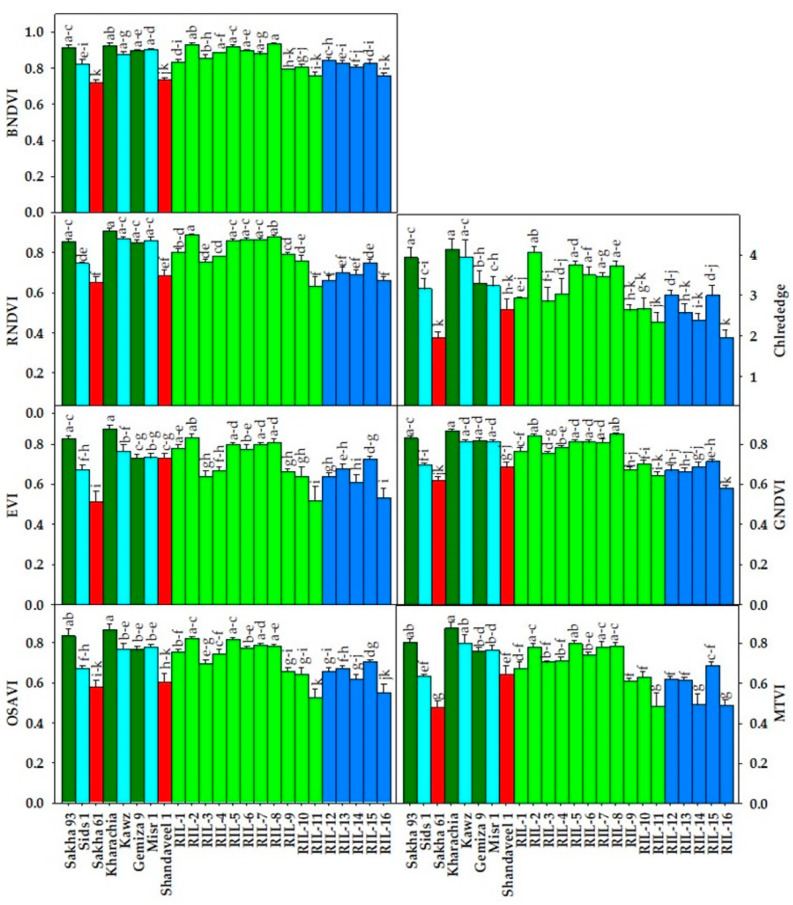
Genotypic variability in seven vegetation spectral reflectance indices among 24 RILs/cultivars under salinity conditions. The full names of indices are mentioned in Table 6 in the Materials and Methods Section. Bars with the same letters are not significantly different at the 0.05 level based on Tukey’s test. Data for each trait are the average of two seasons and three replicates, with bars representing the standard error (n = 6). The dark green color represents salt-tolerant genotypes; the red color represents salt-sensitive genotypes; the aqua color indicates moderately tolerant genotypes; the light green color represents 11 RILs resulting from the cross between salt-tolerant (Sakha 93) and salt-sensitive (Sakha 61) genotypes; and the blue color represents 5 RILs resulting from the cross between salt-tolerant and moderately salt-tolerant (Sids 1) genotypes.

**Figure 3:**
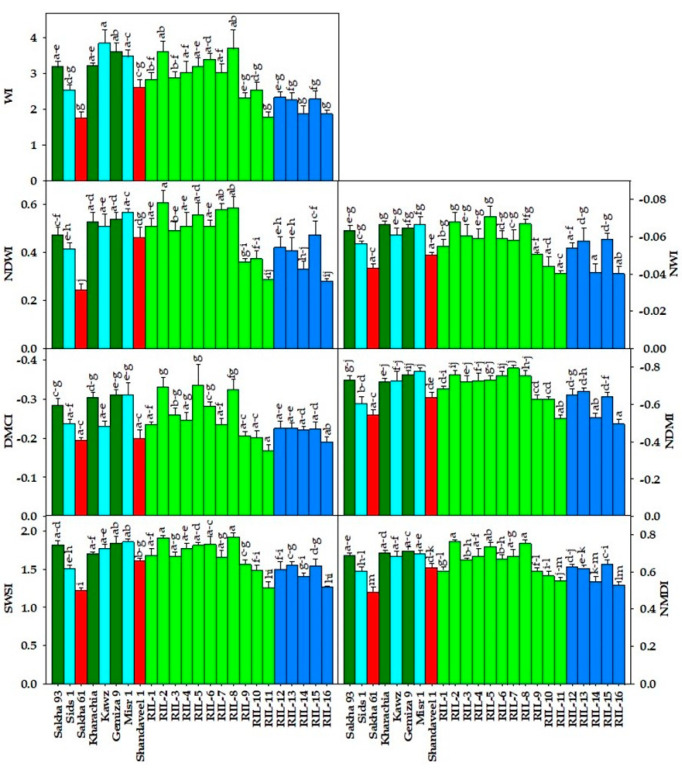
Genotypic variability in seven water spectral reflectance indices among 24 RILs/cultivars under salinity conditions. The full names of indices are mentioned in Table 6 in the Materials and Methods Section. Bars with the same letters are not significantly different at the 0.05 level based on Tukey’s test. Data for each trait are the average of two seasons and three replicates, with bars representing the standard error (n = 6). The dark green color represents salt-tolerant genotypes; the red color represents salt-sensitive genotypes; the aqua color indicates moderately tolerant genotypes; the light green color represents 11 RILs resulting from the cross between salt-tolerant (Sakha 93) and salt-sensitive (Sakha 61) genotypes; and the blue color represents 5 RILs resulting from the cross between salt-tolerant and moderately salt-tolerant (Sids 1) genotypes.

Figure 1, Figure 2 and Figure 3 show the variability among genotypes for four destructively measured morpho-physiological traits (Figure 1), seven vegetation SRIs (Figure 2), and seven water SRIs (Figure 3). The tested RILs/cultivars showed a wide range in both measured destructive traits and various SRIs, which is an interesting observation. For instance, the mean values of LRWC, Chlt, plant dry weight (PDW), and grain yield (GY) across two seasons and all RILs/cultivars ranged from 59.0 to 76.7%, 1.00 to 2.84 mg g^−1^ FW, 4.28 to 6.40 g plant^−1^, and 2.74 to 4.79 ton ha^−1^, respectively (Figure 1). The vegetation SRIs and water SRIs showed also a wide range among RILs/cultivars. For vegetation SRIs, the values for the blue normalized difference vegetation index (BNDVI), green normalized difference vegetation index (GNDVI), red normalized difference vegetation index (RNDVI), red-edge chlorophyll index (Chl_red-edge_), enhanced vegetation index (EVI), modified transformed vegetation index (MTVI), and optimized soil adjusted vegetation index (OSAVI) ranged from 0.72 to 0.93, 0.58 to 0.87, 0.66 to 0.91, 1.94 to 4.12, 0.51 to 0.87, 0.48 to 0.87, and 0.43 to 0.86, respectively, across seasons and RILs/cultivars (Figure 2). For water SRIs, the values for the ratio water index (RWI), normalized water index (NWI), normalized difference water index (NDWI), normalized difference moisture index (NDMI), dry matter content index (DMCI), normalized multi-band drought index (NMDI), and salinity and water stress index (SWSI) ranged from 1.75 to 3.85, −0.071 to −0.40, 0.24 to 0.61, −0.80 to −0.50, −0.34 to −0.17, 0.50 to 0.76, and 1.22 to 1.92 across seasons and RILs/cultivars (Figure 3). These ranges indicate that the maximum values of vegetation SRIs and water SRIs were 1.5 to 2 times higher than the minimum values, similar to destructive traits. This confirms that there were broad genotypic differences for vegetation SRIs and water SRIs, similar to the destructive traits. Interestingly, the salt-tolerant genotypes Kharachia-65 and Sakha 93 were among the top five genotypes with the highest values for morpho-physiological traits and different SRIs. In contrast, the salt-sensitive genotype Sakha 61 ranked among the bottom five genotypes with the lowest values for both traits and SRIs (Figure 1, Figure 2 and Figure 3). Additionally, among the eleven RILs derived from the cross between Sakha 93 and Sakha 61, two (RIL2 and RIL8) exhibited similar or superior values for both traits and SRIs when compared to the two salt-tolerant genotypes Sakha 93 and Kharachia-65. The RIL11 from the first crossing and RIL16 from the second crossing exhibited similar or lower values for both traits and SRIs compared to the salt-sensitive genotype Sakha 61. Gemmeza 9 displayed comparable values for most traits and SRIs to the salt-tolerant genotypes Sakha 93 and Kharachia-65, while Shandaweel-1 displayed comparable values to the salt-sensitive genotypes Sakha 61 (Figure 1, Figure 2 and Figure 3).

### 2.3. Comparison of Genotypic Parameters for Morpho-Physiological Traits and Different SRIs

Table 2 compares genetic parameters for variability among morpho-physiological traits and various SRIs. The broad-sense heritability (h^2^) of most SRIs was comparable to that of agro-physiological traits. The h^2^ values for morpho-physiological traits, vegetation SRIs, and water SRIs ranged from 48.5% to 84.0%, 57.2% to 78.7%, and 21.3% to 78.3%, respectively. The DMCI was the only index among the different SRIs that had the lowest h^2^ value (21.3%), followed by the LRWC trait (48.5%). The h^2^ values for Chlt (61.8%), the BNDVI (59.9%), the Chl_red-edge_ (57.2%), and the WI (51.1%) are moderate, while other traits and SRIs have h^2^ values exceeding 65.0% (Table 2).

In general, the genotypic coefficient of variability (GCV) and phenotypic coefficient of variability (PCV) of most SRIs were comparable to those of morpho-physiological traits. Additionally, the values of the GCV were closer to those corresponding to the PCV for most morpho-physiological traits and various SRIs (Table 2). Among the morpho-physiological traits, LRWC had the lowest GCV (1.35) and PCV (1.94) values, while Chlt showed the highest values (7.26 and 9.23, respectively). The Chl_red-edge_ (a vegetation SRI) along with the RWI, DMCI, and SWSI (water SRIs) had the highest values for the GCV (7.8%, 7.8%, 4.2%, and 7.28%) and PCV (10.3%, 10.9%, 9.2%, and 8.75), respectively, which were comparable to the values of the Chlt trait. Additionally, the PCV values were clearly higher (1.3 to 2.2 times) than the GCV values for the LRWC, Chlt, Chl_red-edge_, RWI, and DMCI compared to other traits and SRIs. Interestingly, the PCV and GCV values for various SRIs were two to five times higher than LRWC values. Additionally, the PCV and GCV values for most SRIs were comparable with those of PDW and GY traits (Table 2).

The lowest genetic gain (GG) was observed in LRWC (1.94%) compared to PDW (7.78%), Chlt (11.76%), and GY (10.32%). Notably, the GG values for most vegetation SRIs (6.75% to 12.14%) and water SRIs (6.81% to 12.48%) were comparable with those of the last three morpho-physiological traits (Table 2).

### 2.4. Genotypic Classification According to Morpho-Physiological Traits and Different SRIs

**Figure 4:**
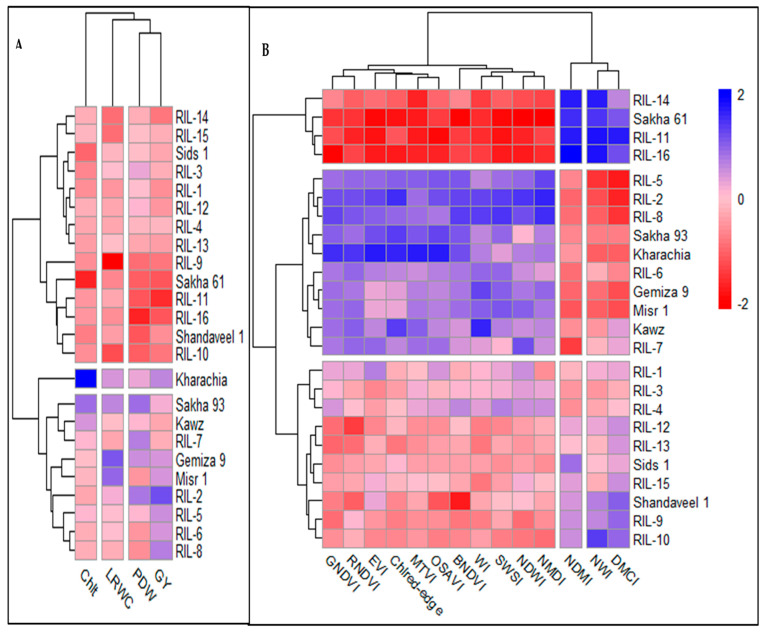
Heatmap cluster analysis displaying the associations among 24 wheat RILs/cultivars based on four destructively measured traits (**A**) and different spectral reflectance indices (SRIs) (**B**). The different color schemes and densities were adjusted based on associations between RILs/cultivars, morpho-physiological traits, and SRIs. The darker red indicates lower values, while the darker blue indicates higher values. PDW, LRWC, Chlt, and GY indicate plant dry weight (g plant^−1^), leaf relative water content (%), total chlorophyll content (mg g^−1^ FW), and grain yield (ton ha^−1^), respectively. The full names of the abbreviations for the different SRIs are listed in Table 6 in the Materials and Methods Section.

The hierarchical clustering heatmaps (HCHs) were used to group RILs/cultivars based on morpho-physiological traits and different SRIs separately (Figure 4). This analysis offered valuable insights into the phenotypic similarities and differences among the tested RILs/cultivars. It is worth noting that the HCH based on different SRIs divided the tested RILs/cultivars into three main groups. They successfully distinguished salt-tolerant genotypes from sensitive ones, similar to the HCH based on morpho-physiological traits. Both HCHs grouped the salt-sensitive genotype Sakha 61 and the three bottom RILs (11, 14, and 16) together. This group exhibited lower values for morpho-physiological traits, vegetation SRIs, and four water SRIs (RWI, NDWI, NDMI, and SWSI), as well as higher values for the remaining three water SRIs (NWI, DMCI, and NMDI). The HCH analysis, based on morpho-physiological traits, separated the salt-tolerant genotype Kharachia-65 into a distinct group. It grouped the other salt-tolerant genotype Sakha 93 and the top five RILs (2, 5, 6, 7, and 8) together in another group. Both groups showed higher values for morpho-physiological traits, vegetation SRIs, and four water SRIs (RWI, NDWI, NDMI, and SWSI), as well as lower values for the remaining three water SRIs (NWI, DMCI, and NMDI). The HCH analysis based on different SRIs collected both salt-tolerant genotypes and the top five RILs in one group, exhibiting similar characteristics to the previous two groups (Figure 4). Both HCHs grouped the Gemiza 9, Misr 1, and Kawz cultivars together with the salt-tolerant genotype Sakha 93, suggesting that these cultivars can be considered as salt-tolerant genotypes. The HCH based on morpho-physiological traits grouped the Sids 1 and Shendaweel-1 cultivars together with the salt-sensitive genotype Sakha 61. However, when using HCHs based on different SRIs, they were separated into distinct groups. This suggests that both cultivars can be considered salt-sensitive based on morpho-physiological traits, but moderately salt-sensitive based on SRIs (Figure 4). Finally, the HCH based on different SRIs successfully grouped the tested RILs/cultivars with similar phenotypic alterations together, similar to the HCH based on morpho-physiological traits.

The results of the HCH based on SRIs also show that the HCH grouped all vegetation SRIs and four water SRIs (RWI, NDWI, NDMI, and SWSI) together in one cluster, with high values for these SRIs in salt-tolerant genotypes. The remaining three water SRIs (NWI, DMCI, and NMDI) were split into two separate clusters, with high values for these three SRIs in salt-sensitive genotypes and low values in salt-tolerant genotypes (Figure 4). Similarly, the HCH grouped morpho-physiological traits into three distinct groups. Morphological traits (PDW and GY) were grouped together. The salt-tolerant genotype Kharachia-65, which separated into a single group, exhibited higher values for Chlt than other genotypes. The cultivars and RILs identified as salt-tolerant genotypes showed high values for LRWC, PDW, and GY. Conversely, the cultivars and RILs identified as salt-sensitive genotypes had lower values for all four morpho-physiological traits (Figure 4).

### 2.5. Genotypic Classification According to SSR Markers

**Figure 5:**
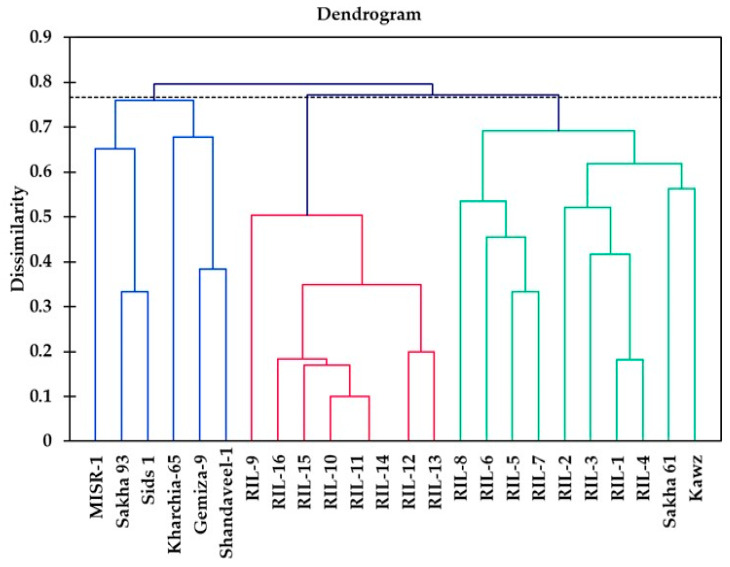
Unweighted neighbor-joining clustering trees of 24 RILs/cultivars based on 34 simple sequence repeat (SSR) markers linked to salt-tolerant genes. The blue lines represent the salt tolerant group, red lines represent moderately salt-tolerant group, and green lines showed the salt sensitive group.

Based on the allelic data from 34 SSR markers associated with salinity tolerance, the 24 wheat RILs/cultivars were grouped into three clusters (Figure 5). Notably, the 34 SSR markers grouped the two salt-tolerant genotypes Sakha 93 and Kharachia-65 together in one cluster while separating the salt-sensitive check genotype Sakha 61 into a different cluster. Furthermore, they also successfully grouped Gemiza-9 and Misr-1 together with Sakha 93 in one cluster, consistent with the HCH based on morpho-physiological traits and different SRIs. Additionally, the moderately salt-tolerant genotypes Sids 1 and Shandaweel-1 were initially grouped with Sakha 61 based on morpho-physiological traits but were separated from Sakha 61 in another cluster based on different SRIs. However, when clustering genotypes based on 34 SSR markers, Sids 1 and Shandaweel-1 were grouped in the same cluster along with the salt-tolerant genotypes Sakha 93 and Kharachia-65 (Figure 5).

The clustering pattern of 24 wheat genotypes/RILs based on either morpho-physiological traits or different SRIs (phenotypic classification) was compared to the clustering based on 25 SSR markers linked to salt-tolerant genes (genotypic classification). The Mantel test showed a significant positive correlation between the clustering of 24 wheat genotypes/RILs based on morpho-physiological traits and SSR data (r = 0.195, *p* < 0.0003), indicating a moderate similarity between the phenotypic clustering using destructively measured traits and SSR-based clusters. Furthermore, a significant positive correlation was found between the clustering based on different SRIs and SSR data-based clusters.

### 2.6. Association of SSR Markers with Destructively Measured Morpho-Physiological Traits and Different SRIs

Table 3 displays the key SSR markers that show a significant association with morpho-physiological traits and SRIs, as determined using a stepwise linear regression analysis. Notably, the SSR markers showed a strong association with vegetative SRIs (R^2^ ranged from 0.66 to 0.86) and water SRIs (R^2^ ranged from 0.56 to 0.97), similar to morpho-physiological traits (R^2^ ranged from 0.62 to 0.75), except for Chlt, which had a moderate association with SSR markers (R^2^ = 0.39). The SSR markers had a strong association with PDW (R^2^ = 0.75), followed by GY (R^2^ = 0.66) and LRWC (R^2^ = 0.62). They also showed a moderate association with Chlt (R^2^ = 0.39). Additionally, the SSR markers exhibited a strong association with all vegetation SRIs (R^2^ ≥ 0.66) and water SRIs (R^2^ ≥ 0.67), except for the NWI (R^2^ = 0.56) (Table 3).

Among the SSR markers selected using stepwise linear regression, the SSR marker Barc44 exhibited the highest variations in LRWC (R^2^ = 0.36), GY (R^2^ = 0.37), the GNDVI (R^2^ = 0.49), MTVI (R^2^ = 0.42), RWI (R^2^ = 0.51), NWI (R^2^ = 0.40), NDWI (R^2^ = 0.42), SWSI (R^2^ = 0.42), and NDMI (R^2^ = 0.43). The marker Gmw335 showed the most variations in the RNDVI (R^2^ = 0.41), Chl_red-edge_ (R^2^ = 0.37), and EVI (R^2^ = 0.39), while Gm350 accounted for the most variations in the BNDVI (R^2^ = 0.30) and NDMI (R^2^ = 0.33). Additionally, markers Wmc245, Gwm55, and Cfd9 exhibited the most variations in Chlt (R^2^ = 0.24), PDW (R^2^ = 0.26), and the OSAVI (R^2^ = 0.56), respectively (Table 3). The marker Cfd9 explained 15%, 15%, 19%, 18%, 24%, 27%, 23%, 21%, 30%, 16%, 14%, and 27% of the total variations in Chlt, GY, GNDVI, RNDVI, Chl_red-edge_, EVI, MTVI, RWI, NDWI, NWI, DMCI, and SWSI, respectively. The markers Wmc154, Wmc11, Barc34, Wmc245, Barc167, Barc34, Wmc154, and Wmc 405 explained 18%, 18%, 14%, 29%, 14%, 16%, 21%, and 19% of the total variations in PDW, LRWC, GY, the BNDVI, RNDVI, DMCI, NMDI, and NDMI, respectively (Table 3).

### 2.7. Hyperspectral Assessment of Destructively Measured Morpho-Physiological Traits

This study also aimed to assess if spectral reflectance tools could expedite the phenotyping process by providing a quicker alternative to conventional methods for assessing morpho-physiological traits. Data from 24 RILs/cultivars collected over two years were used to establish linear and non-linear correlations between various SRIs and the values of each morpho-physiological trait obtained through destructive measurements (Table 4).

The vegetation SRIs and water SRIs showed a strong linear relationship with GY, with R^2^ values ranging from 0.69 to 0.80 and 0.69 to 0.90, respectively. The vegetation SRIs had a strong to moderate linear relationship with PDW, with R^2^ values ranging from 0.37 to 0.60. The water SRIs also showed a moderate linear relationship with PDW, with R^2^ values ranging from 0.38 to 0.50, except for the RWI and DMCI, which had a moderate (R^2^ = 0.40) and strong (R^2^ = 0.56) second-order relationship with PDW (Table 4). The vegetation SRIs (R^2^ ranged from 0.40 to 0.68) outperformed the water SRIs (R^2^ ranged from 0.19 to 0.28) in estimating Chlt content. All vegetation SRIs exhibited a second-order relationship with Chlt, except for the BNDVI and Chl_red-edge_, while all water SRIs showed a linear relationship with Chlt. Notably, the Chl_red-edge_, EVI, MTVI, and OSAVI showed a strong relationship (R^2^ ranged from 0.53 to 0.68) with Chlt, surpassing the performance of other SRIs. The vegetation SRIs and water SRIs provided comparable estimates of the LRWC with moderate relationships (R^2^ values ranged from 0.23 to 0.42 and 0.30 to 0.44, respectively). All vegetation SRIs, except the Chl_red-edge_, had a second-order relationship with LRWC, while all water SRIs, except the NDMI and SWSI, had a linear relationship (Table 4).

### 2.8. Principal Component Analysis of Morpho-Physiological Traits and Different SRIs

**Figure 6:**
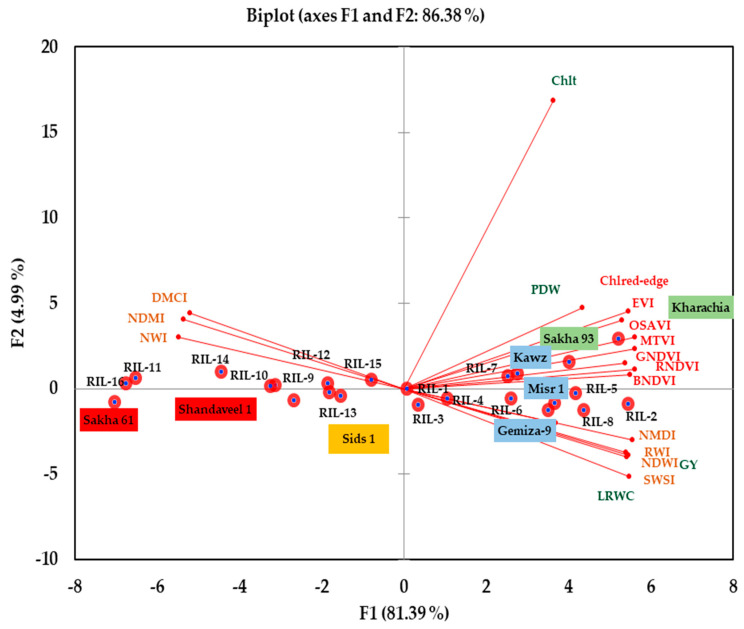
Principal component analysis biplot for 24 RILs/cultivars, morpho-physiological traits, vegetation SRIs, and water SRIs. PDW, LRWC, Chlt, and GY indicate plant dry weight (g plant^−1^), leaf relative water content (%), total chlorophyll content (mg g^−1^ FW), and grain yield (ton ha^−1^), respectively. The full names of the abbreviations for the different SRIs are listed in Table 6 in the Materials and Methods Section.

A principal component analysis (PCA) was performed on morpho-physiological traits, different SRIs, RILs/cultivars, and seasons together (Figure 6). The first (PC1) and second (PC2) components accounted for 81.39% and 4.99% of the total variation in all traits and SRIs, respectively.

The PCA grouped morpho-physiological traits and SRIs into two main groups. Group 1 included morpho-physiological traits, vegetation SRIs, and four water SRIs (RWI, NDWI, NDMI, and SWSI). This group correlated strongly with PC1 and was closely associated with the salt-tolerant genotypes Sakha 93 and Kharachia-65, as well as the top five RILs with high values for these traits and SRIs. Group 2 included the remaining three water SRIs (NWI, DMCI, and NMDI). This group exhibited a significant negative correlation with PC2 and was closely linked to the salt-sensitive genotype Sakha 61, as well as the bottom five RILs with lower values for morpho-physiological traits and most SRIs. Additionally, the vectors of morpho-physiological traits and SRIs of group 1 formed an acute angle between each other, indicating a positive association. In contrast, the vector of the three water SRIs of group 2 formed a straight angle with the vectors of group 1, indicating a negative association between the two groups (Figure 6).

### 2.9. Assessment of Morpho-Physiological Traits Using a Combination of SRIs and PLSR Models

Table 5 presents a summary of the calibration (cal.) and validation (val.) datasets utilized in the PLSR models for estimating morpho-physiological traits with all SRIs. Overall, the PLSR models accurately estimated all morpho-physiological traits in both the cal. (R^2^ _Cal._ ranged from 0.72 to 0.96) and val. (R^2^ _Val._ ranged from 0.66 to 0.94) datasets (Table 5). The PLSR model with three SRIs (OSAVI, NMDI, and DMCI) was identified as the most effective to accurately estimate GY. This model demonstrated a strong performance with R^2^ values of 0.96 and 0.94 for the training and validation datasets, respectively. The PLSR models using the MTVI for Chlt and the BNDVI for LRWC proved to be successful in accurately estimating Chlt and LRWC, achieving R^2^ values of 0.81 and 0.85 for the training dataset and 0.75 and 0.68 for the validation dataset, respectively. The PLSR model with the RNDVI and OSAVI was identified as the most effective for estimating PDW, with R^2^ values of 0.72 and 0.66 for the training and validation datasets, respectively (Table 5).

## 3. Discussion

Salinity is a significant threat to wheat sustainability in arid and semiarid regions, where brackish water is commonly used for irrigation and the salinity-affected soil is expanding over time. Therefore, developing salt-tolerant wheat varieties is a cost-effective solution to ensure the sustainability of this crop in these regions. In the last two decades, there have been considerable efforts to improve the salt tolerance of wheat genotypes, but progress has been limited. Reviews of current research have pointed out challenges in creating salt-tolerant wheat genotypes through breeding programs. These obstacles include the lack of reliable screening criteria, limited genetic diversity for salt tolerance, the inadequate assessment of salt tolerance in real field conditions, and the inefficiency of traditional phenotyping methods due to time and cost limitations when assessing the salt tolerance of a large number of genotypes [14,15,21,45,46]. In this study, we assessed 24 wheat genotypes, including salt-tolerant and salt-sensitive check genotypes, as well as sixteen F_8_ RILs, to cover a broad spectrum of genetic diversity for salinity tolerance. The ANOVA supported this objective and revealed significant variations among genotypes for four morpho-physiological traits (PDW, LRWC, Chlt, and GY) in each year and when the data from two years were combined (Table 1). These findings also highlight the importance of these four traits as screening criteria for evaluating the salt tolerance of wheat genotypes, which will be discussed further below. Importantly, the salt tolerance of these genotypes was assessed in real field conditions using both destructively measured morpho-physiological traits and high-throughput genotyping and phenotyping tools that are cost-effective, time-efficient, and non-destructive.

Salt stress components, such as osmotic stress, nutritional deficits for essential ions, and specific ion toxicities, interact to restrict several physiological and biochemical processes involved in plant growth and development. These negative impacts of salinity stress ultimately translate into significant decreases in plant biomass. Furthermore, plant biomass, which encompasses a range of physiological processes and integrates plant responses to salinity stress across different growth stages, is closely linked to radiation use efficiency, the conversion of light into biomass, and overall crop yield. Additionally, improving grain yield through genetic improvements may require a focus on increasing plant biomass rather than the harvest index. Importantly, when plants are exposed to high salinity levels, their energy and metabolic resources are diverted to activate stress-tolerance mechanisms instead of being used for growth and biomass production [6,40,47,48,49]. These facts about plant biomass indicate that this plant trait could serve as a reliable screening criterion for distinguishing salt tolerance among various wheat genotypes. The study results confirmed that PDW measured at 90 days after sowing is a crucial screening criterion for assessing salt tolerance in the tested genotypes. Significant differences (*p* < 0.001) were observed in PDW among the genotypes (Table 1 and Figure 1). The PDW also exhibited high broad-sense heritability (h^2^ = 77.52%) and a moderate value for GG (7.8%), with the PCV value comparable to the GCV value (Table 2). Notably, SSR markers showed a strong association with PDW (R^2^ = 0.75), with markers Gwm55 and Wmc154 displaying a significant association (R^2^ = 0.64) with this trait (Table 3). These findings indicate that the PDW is under genetic control, emphasizing the importance of phenotyping this trait as an important screening criterion for evaluating salt tolerance in wheat genotypes under real field conditions.

Previous studies have demonstrated that the water status of plants is essential for efficient photosynthesis. When leaves have reduced turgor potential, it can affect their enlargement, stomata opening, and consequently, the overall efficiency of photosynthesis in plants [6,50]. High levels of NaCl in the soil can hinder a plant’s water absorption, leading to physiological drought stress. This affects the plant’s water balance, particularly its LRWC, which reflects the balance between water uptake and transpiration. Therefore, the LRWC can serve as a reliable indicator of abiotic stress compared to other plant physiological and biochemical characteristics. It is also closely linked to other plant water status parameters such as water potential, osmotic potential, and turgor pressure [51]. These facts about LRWC indicate that this trait may be considered an appropriate phenotypic screening criterion for evaluating the salt tolerance of wheat genotypes. In this study, the LRWC showed significant differences (*p* < 0.001) among tested genotypes (Table 1) and exhibited a moderate heritability in a broad sense (h^2^ = 48.51%) (Table 2). On the other hand, SSR markers showed a strong association with LRWC (R^2^ = 0.62), with markers Barc44 and Wmc11 displaying a significant variation (R^2^ = 0.54) in this trait (Table 3). These findings indicate that LRWC could serve as a useful screening criterion for identifying salt-tolerant genotypes at both phenotypic and genotypic levels.

The result of this study also found that the Chlt showed significant differences (*p* < 0.001) among tested genotypes (Table 1 and Figure 1) with a high heritability in a broad sense (h^2^ = 61.84%) and a moderate GG (11.76%) (Table 2). The results for Chlt, both phenotypic and genotypic, confirm its significance as a key screening criterion for assessing salt tolerance in wheat genotypes under actual field conditions. This observation is likely because the reduction in chlorophyll content is a typical response to salinity stress, with salt-sensitive genotypes showing a more significant decrease compared to salt-tolerant ones [6,52]. The decrease in chlorophyll content under salinity conditions may be caused by the combined osmotic and ionic toxicity of salinity stress. This can lead to increased activity of chlorophyll-degrading enzymes, such as reactive oxygen species (ROS), as well as the disruption of enzymes involved in chlorophyll synthesis. Ultimately, this results in reduced chlorophyll formation or accelerated chlorophyll degradation [53]. However, other studies have indicated that salinity stress has a lesser effect on chlorophyll content, with no significant differences observed between salinity-treated and control groups. This is attributed to the development of smaller, thicker leaves with a higher concentration of chloroplasts per leaf area under salinity stress. Consequently, there is an increase in chlorophyll content per unit area [6,54]. This indicates that the chlorophyll content may not remain stable if salinity stress has a significant impact on leaf anatomy, such as the size and thickness of the leaf. This could explain why SSR markers only showed a weak relationship with chlorophyll content (R^2^ = 0.39), as shown in Table 3.

In general, plant breeders usually prioritize the final grain yield (GY) when assessing genotypes under abiotic stress. This is because GY reflects various crucial factors that develop at key growth stages during the crop growth cycle, such as biomass allocation, sunlight interception, and conversion. This makes GY a comprehensive indicator of genotype performance and stress tolerance [15,46,55]. Consequently, GY can serve as a valuable screening criterion for evaluating the salt tolerance of genotypes. The results of this study confirm this statement and found that GY showed highly significant differences (*p* < 0.001) among genotypes (Table 1 and Figure 1), with a high broad-sense heritability of 84.0% and a moderate GC of 10.32%. The PCV was also comparable to the GCV. Additionally, three SSR markers (Barc44, Cfd9, and Barc34) explained 66% of the variations in GY. These results confirm the importance of GY as a screening criterion for evaluating the salt tolerance of wheat genotypes.

The effectiveness of four morpho-physiological traits in distinguishing salt-tolerant genotypes from sensitive ones was also validated using HCHs. The traits successfully differentiated the salt-tolerant genotypes Sakha 93 and Kharachia-65 from the salt-sensitive genotype Sakha 61 (Figure 4A). Additionally, the Mantel test revealed a significant positive correlation between the phenotypic clustering based on these traits and SSR-based clusters (r = 0.195, *p* < 0.0003). These findings fully confirmed the importance of the four traits as screening criteria for evaluating salt tolerance in wheat genotypes. However, conventional phenotyping for these traits is often laborious and time-consuming. Therefore, in the next section, we delve into the use of a hyperspectral canopy reflectance tool to assess the salt tolerance of wheat genotypes. This method allows for the quick and non-invasive phenotyping of large germplasm collections. By efficiently assessing important morpho-physiological traits in a cost-effective manner, it simplifies the process of selecting genotypes without compromising the precision of conventional phenotyping methods.

### Ability of Different Spectral Reflectance Indices as an Alternative Screening Criteria for Evaluating Salt Tolerance in Wheat Genotypes

In general, salinity stress causes significant changes in various biophysical and biochemical properties of plant canopies, resulting in alterations in their spectral signatures across the entire spectrum (400–2500 nm). These changes can be detected through various spectral regions. For instance, variations in leaf pigment content, plant health, and photosynthetic capacity are noticeable in the VIS (400–700 nm) spectrum. Changes in biomass accumulation, leaf structure, and leaf area index impact canopy reflectance in the NIR (7900–1300 nm) region. Changes in plant water status can be detected through specific water absorption bands in the NIR and SWIR (1300–2500 nm) regions. To capture these changes in a simple way, two types of SRIs have been developed. The first type, vegetation SRIs, includes wavelengths from the VIS, red-edge (700–850 nm), and NIR regions. These indices are effective in detecting variations in chlorophyll pigments, vegetative vigor, photosynthetic efficiency, and biomass accumulation. The second type, water SRIs, incorporates weak and strong water absorption bands from the NIR and SWIR regions, making them suitable for detecting changes in plant water status [24,28,29,30,33,34,56,57]. This close relationship between SRIs and plant characteristics indicates that SRIs can be used as non-destructive and cost-effective screening criteria for evaluating salt tolerance in wheat genotypes. This offers an alternative to traditional morpho-physiological traits commonly used in breeding programs to evaluate salt tolerance. Moreover, SRIs can be a viable substitute for traditional phenotyping methods in indirectly assessing the performance of wheat genotypes under salinity stress conditions. To investigate the ability of SRIs as alternative screening criteria, a genetic analysis, heritability, cluster analysis, and association with SSR markers were tested for SRIs and compared with those of morpho-physiological traits. SRIs can be used as alternative screening criteria to evaluate salt tolerance if they are comparable to direct destructive traits in terms of genetic analysis and heritability. In general, to make reliable selections based on additive gene action, it is recommended to consider broad-sense heritability, the GCV, and GA together. Johnson et al. [58] emphasized the importance of evaluating both heritability and GG, as high heritability does not always translate to a high GG. The results of this study found highly significant differences among genotypes in different SRIs (*p* < 0.001) (Table 1 and Figure 2 and Figure 3). Most SRIs had high heritability and a moderate GG, with similar values for the GCV and PCV (Table 2). The SRIs effectively clustered check salt-tolerant genotypes Sakha 93 and Kharachia-65 together and separated check salt-sensitive genotype Sakha 61 (Figure 4B). SSR markers showed a strong association with SRIs (R^2^ ranged from 0.56 to 0.89), with specific markers (Barc44, Gwm350, Gwm 335, and Cfd9) displaying the most variation in SRIs (Table 3). Importantly, the Mantel test revealed a significant positive correlation between the phenotypic clustering based on different SRIs and SSR-based clusters (r = 0.269, *p* < 0.0001). The heritability and genetic gain results for various SRIs indicate that genetic factors play a significant role in the variation in these indices among genotypes. This suggests that phenotypic selection can effectively estimate these SRIs and their phenotypic performance could be valuable for selection in the context of genetic improvement [59,60]. Therefore, SRIs can serve as a dependable screening criterion for evaluating salt tolerance in wheat genotypes, eliminating the requirement for destructive morpho-physiological traits. Mohi-Ud-Din et al. [19], Gutierrez et al. [33], El-Hendawy et al. [35], Prasad et al. [61], and Babar et al. [62] found similar results under drought stress conditions. They reported that significant genetic gains could be achieved by incorporating SRIs’ measurements during selection in wheat breeding programs, especially when the selection is carried out in mid- and late-breeding generations (F5 to F7).

In this study, we also explore the potential of using SRIs as a substitute for conventional phenotyping techniques to indirectly assess the four morpho-physiological traits. This will help us quickly and non-invasively evaluate the performance of multiple genotypes under salinity stress conditions. Several studies have used SRIs as effective tools to indirectly estimate various plant traits such as chlorophyll content, plant biomass, RWC, and GY in wheat and other cereal crops [1,18,19,33,35,63,64,65,66]. However, most of these studies were conducted under normal growing conditions with a wide range of phenotypic variability in genotypes or drought stress conditions. To our knowledge, only a few studies have investigated the performance of SRIs for indirectly estimating different morpho-physiological traits under salinity stress conditions.

Previous studies have demonstrated that different vegetation SRIs and water SRIs can accurately predict a significant portion of the variability in GY and other plant traits such as plant biomass, chlorophyll content, and water content across various crops and environments [26,33,35,56,57,66,67]. However, there is a discrepancy in studies regarding the effectiveness of vegetation SRIs versus water SRIs in estimating different plant traits. Some studies indicate that water SRIs, incorporating wavelengths from NIR and SWIR regions, are better at capturing genotypic variability in GY and plant biomass than vegetation SRIs, which use VIS, red-edge, and NIR wavelengths, under diverse environmental conditions [33,61,62]. However, other studies show that vegetation SRIs perform better or equally well compared to water SRIs in estimating the GY of spring wheat under water deficit stress conditions [35,67,68,69]. This study found that both types of SRIs were effective in assessing PDW and GY under salinity stress conditions, with the vegetation SRIs and water SRIs showing a moderate relationship with PDW (R^2^ ranged from 0.46 to 0.60 and 0.38 to 0.56) and a strong relationship with GY (R^2^ ranged from 0.69 to 0.80 and 0.69 to 0.90), respectively (Table 4). These results indicate that genotypic variations in plant biomass and GY under salinity conditions can be assessed during the vegetative stage using vegetation SRIs and water SRIs without the need to wait for plants to reach maturity. This suggests that SRIs can be a rapid and straightforward phenotyping tool to effectively assess the salinity tolerance in larger populations in breeding programs within a shorter time frame. The common negative effects of salinity stress, such as reduced biomass, chlorophyll degradation, and changes in leaf structure and water content, may explain why both vegetation SRIs and water SRIs incorporating green, red, red-edge, NIR, and SWIR bands were successful in explaining variations in PDW and GY among tested genotypes in this study.

The study revealed that vegetation SRIs (R^2^ = 0.40–0.68) provided more accurate estimates of Chlt compared to water SRIs (R^2^ = 0.19–0.28). Among the vegetation SRIs examined, the MTVI and OSAVI were the most precise (R^2^ = 0.68 and 0.62, respectively) in estimating Chlt, followed by the Chl_red-edge_ and EVI (R^2^ = 0.53 for both) (Table 4). Similarly, previous studies have demonstrated that SRIs incorporating wavelengths in the red, red-edge, and NIR regions are more reliable indicators of leaf chlorophyll content than indices in other regions. This is because these wavelengths are effective at detecting the absorption characteristics of photosynthetic pigments like chlorophyll-a and -b, α- and β-carotenes, and lutein, which are affected by salinity stress [70,71,72,73]. The superior performance of the MTVI, OSAVI, and EVI in estimating Chlt content in wheat compared to other vegetation SRIs may be attributed to these indices effectively addressing saturation issues across a range of LAI values from two to eight and taking into account internal and surface structural effects on leaf surface reflectance. On the other hand, other vegetation SRIs like the BNDVI, GNDVI, and RNDVI are less effective in estimating chlorophyll content, as they may reach saturation levels when LAI values are higher than two [72,73,74,75,76,77].

The study found that both vegetation SRIs and water SRIs are equally effective in estimating LRWC and showed a moderate relationship (R^2^ = 0.23–0.42 and 0.30–0.44, respectively) with this plant trait (Table 4). The water bands in the NIR and SWIR regions are known to be effective for detecting the water status of plants. The water absorption bands in NIR region, particularly at 970 nm, can penetrate deeper into the canopy, allowing for the estimation of plant water status [33]. Moreover, the water bands in the SWIR regions such as 1400, 1450, 1650, 1920, 1950, and 2250 nm are sensitive to changes in plant water status and less affected by noise from internal leaf structure [78]. This may explain why the water SRIs that combine NIR and SWIR wavelengths were successful in estimating LRWC under salinity conditions in this study. The study’s findings indicate that variations in LRWC could also be successfully estimated by vegetation SRIs that incorporate wavelengths related to pigment levels, photosynthetic capacity, growth vigor, structural leaf compounds, leaf cellular structure, and biomass accumulation rather than being influenced by alterations in plant water content. Kovar et al. [79] found similar results, indicating that the green wavelength in the VIS spectrum was effective in estimating the plant water status, which was expressed by the relative water content, leaf water potential, and equivalent water thickness of soybean under varying irrigation levels. Under stress conditions, plants experience a decrease in cell turgor due to reduced water content. This leads to cell shrinkage, lower chlorophyll levels, and increased reflection in the green spectral region [80]. Colovic et al. [30] found that some vegetation SRIs incorporating red-edge wavelengths were also effective in estimating changes in water status in sweet maize. This could explain why vegetation SRIs accurately estimated LRWC as water SRIs under salinity stress in the present study.

To gain a better understanding of the relationships between morpho-physiological traits, different SRIs, and genotypes, a PCA was conducted. The results revealed that traits, vegetation SRIs, and four water SRIs (RWI, NDWI, NDMI, and SWSI) were closely linked and associated with salt-tolerant genotypes and the top five RILs exhibiting high values for traits and SRIs. In contrast, the remaining three water SRIs (NWI, DMCI, and NMDI) were grouped separately and were closely linked to a salt-sensitive genotype, specifically the bottom five RILs with lower values for traits and most SRIs (Figure 6). The results indicate that both vegetation SRIs and water SRIs are successful in differentiating between salt-tolerant and salt-sensitive genotypes, similar to traditional morpho-physiological traits that are measured destructively. The PCA further validated that various SRIs can be effective, non-destructive, and affordable tools for phenotyping in wheat breeding programs under salinity stress conditions, particularly as these SRIs varied significantly among genotypes and were closely linked to SSR markers.

Due to the limited number of wavelengths included in each SRI, many of them are unable to effectively address the effects of soil background, saturation of the leaf area index, chlorophyll content, and biomass on the performance of the index of interest. This inefficiency hinders the accurate estimation of morpho-physiological traits using individual SRIs [35,73,75]. Additionally, the same set of SRIs exhibits substantial multicollinearity among them, which limits their accuracy in predicting plant traits when dealing with treatments or genotypes that have similar reflectance and absorption patterns. Hence, as previously mentioned in the studies, combining PLSR with multiple SRIs has been shown to improve the accuracy of estimating various plant traits [35,81,82,83]. The findings of this study also validated that the PLSR models utilizing all vegetation and water SRIs improved the accuracy of the estimation of various morpho-physiological traits in both cal. and val. when compared to using individual SRIs (refer to Table 4 and Table 5 for comparison). This improvement could be attributed to the PLSR models in this study incorporating data from 14 SRIs and determining the optimal number of factors to accurately represent the calibration data without overfitting.

## 4. Material and Methods

### 4.1. Plant Materials, Experimental Site, and Growing Conditions

The study used a total of twenty-four different bread wheat genotypes as genetic plant resources. These included sixteen F_8_ RILs and eight varieties. The eight varieties comprised three parent genotypes (salt-tolerant Sakha 93, moderately salt-tolerant Sids-1, and salt-sensitive Sakha 61) and five additional cultivars (a reference salt-tolerant Kharachia-65, salt-sensitive Shandawel-1, moderately salt-sensitive Misr-1, moderately salt-tolerant Gemiza-9, and Kawz). The salt tolerance of the three parents and Kharachia-65 was assessed in pot experiments and saline field conditions in previous studies conducted by El-Hendawy et al. [10,13], Ashraf [84], and Mansour et al. [85]. The RILs were developed at the Faculty of Agriculture, Suez Canal University in Egypt and the College of Food and Agriculture Sciences at King Saud University in Saudi Arabia. Five out of the sixteen RILs were created from a cross between Sakha 93 and Sids 1, while the remaining eleven were from a cross between Sakha 93 and Sakha 61.

Field trials were conducted at the experimental field of the College of Food and Agriculture Sciences, King Saud University in Dierab, Riyadh, Middle Saudi Arabia (latitude: 24°25′ N, longitude: 46°34′ E, elevation: 400 m a.s.l) during the 2019–2020 and 2020–2021 growing seasons. The area has a typical arid climate with very little winter rainfall and dry summers. The data for air temperature and precipitation at the experimental field during the winter growing season are displayed in Supplementary Figure S1. The information was collected from a weather station near the experimental field.

The soil samples collected from the experimental farm at a depth of 0–30 cm before the installation of the experiment had a sandy loam texture, with 56.7% sand, 28.4% silt, and 14.9% clay. These samples also exhibited specific physiochemical characteristics, namely an electrical conductivity (EC) of 1.12 dS m^−1^, a pH of 7.85, organic matter content of 0.46%, bulk density of 1.48 g cm^−3^, and CaCO_3_ content of 29.22%. Additionally, the nitrogen content was 3.98 g kg^−1^, potassium content was 1.67 mg kg^−1^, and phosphorus content was 0.07 mg kg^−1^. The water holding capacity was 18.56% and the permanent wilting threshold was 7.21%.

### 4.2. Experimental Design, Salinity Treatment, and Agronomic Practices

The experiments were conducted using a randomized complete block design (RCBD) with three replications. The sixteen F_8_ RILs and eight varieties were evaluated under high salinity levels (15 dS m^−1^). To prevent salinity stress shock during germination and seedling establishment, the tested genotypes were initially irrigated with normal water for the first 21 days. Subsequently, they were irrigated with artificial saline water containing 15 dS m^−1^ NaCl for the remainder of the experiment. Saline water was applied using a low-pressure surface irrigation system. This system consisted of a plastic water tank with a capacity of 5.0 m^3^ and a main line with a diameter of 76 mm. The saline water was distributed from the tank to each plot through the main line, which divided into sub-main hoses with manual control valves at each plot to ensure uniform water distribution to each plot (Supplementary Figure S2). The artificial saline water was prepared by dissolving 8.8 g of NaCl per liter. The irrigation amount for wheat was calculated by multiplying the reference daily evapotranspiration (ETo) by the crop coefficient (Kc). The ETo was determined using the modified Penman–Monteith equation, and the Kc value for wheat was obtained from FAO-56 [86]. Based on these data, the genotypes were watered with saline water 10 times throughout their growth stages, with a total water volume of 4800 m^3^ per hectare. Furthermore, the salinity level was regularly monitored throughout the growing season in both years by analyzing the electrical conductivity (EC) of soil samples taken from different places within the plot at a depth of 0–60 cm. The results showed that the EC for these soil samples did not exceed 16.3 dS m^−1^ in either year. In the second year, all plots were irrigated with freshwater multiple times prior to sowing to flush out any accumulated salt from the root zone that may have built up during the first year.

The seeds of each RIL/cultivar were sown at a rate of 15 g m^−2^ in four rows spaced 20 cm apart, 1.5 m long, and 50 cm between plots in the fourth week of November during two growing seasons. The RILs/cultivars were fertilized with phosphorus (P), potassium (K), and nitrogen (N) at a rate of 100, 90, and 150 kg ha^−1^ in the form of calcium superphosphate (18.5% P_2_O_5_), potassium chloride (50% K_2_O), and ammonium nitrate (33.5% N), respectively. The full amount of P was applied before sowing, while K was split into two equal doses, with one dose applied before sowing and the second dose at the late booting growth stage. Nitrogen was divided into three equal doses, with the first dose applied before sowing, the second at the late tillering growth stage, and the last one at the late booting growth stage. To ensure the healthy growth of the crop, we also implemented other recommended agronomic practices, such as timely weeding and pest and disease control.

### 4.3. Phenotypic Monitoring

#### 4.3.1. Morpho-Physiological Trait Measurements

At the anthesis growth stage (90 days after sowing), ten plants were randomly selected from each RIL/cultivar in each replicate to record plant dry weight (PDW). The plants were dried in an oven at 75 °C for three days until they reached a constant weight. The PDW was calculated by averaging the dry weight of 10 plants. Additionally, five fully grown leaves were collected from each RIL/cultivar and replicated to measure two physiological traits, the leaf relative water content (LRWC) and total chlorophyll content (Chlt). To measure LRWC, approximately 0.20 cm^2^ sections were cut from five leaves and weighed to determine their fresh weight (FW). The sections were then soaked in distilled water at room temperature (25 °C) in the dark until fully turgid. After removing external water, the sections were weighed to record the turgid weight (TW). The sections were oven-dried at 75 °C for 72 h and weighed to record the dry weight (DW). Finally, the percentage of LRWC was calculated using the following formula:

(1) LRWC=(FW−DW)/(TW−DW)×100

Total chlorophyll content (Chlt) was measured using spectrophotometry. Fresh leaf samples (0.4 g) were extracted from five leaves, washed with distilled water, and homogenized in 5 mL of 80% acetone at room temperature in the dark until they turned completely white. The sap was then centrifuged at 400 rpm for five minutes and adjusted to a final volume of 50 mL with 80% acetone. The pigment extracts were analyzed for absorption spectra using a UV/vis spectrophotometer (UV-2550, Shimadzu, Tokyo, Japan) at wavelengths of 645 nm (A645) and 663 nm (A663). Finally, the Chlt was calculated using the following formula, described by Lichtenthaler and Wellburn [87]:

(2) $$Chlt=\left[\left(20.21\times A645\right)+\left(8.02\times A663\right)\right]$$

At the maturity stage, which took place on 21 April in both growing seasons, a 0.75 m^2^ area from each RIL/cultivar and replicate was manually harvested. The harvested samples were air-dried for one week, threshed to collect grains, cleaned, and their moisture content was adjusted to 14%. The grains were weighed and converted to tons per hectare.

#### 4.3.2. Hyperspectral Reflectance Measurements and Extracted Spectral Reflectance Indices

The canopy’s hyperspectral reflectance was measured at the anthesis growth stage, along with the assessment of morpho-physiological traits. The measurements were taken under cloudless and windless conditions between 10:00 and 14:00 using a portable full-range hyperspectral ASD FieldSpec 4 device (Analytical Spectral Devices Inc., Malvern Panalytical, Malvern, Worcs, UK) (Figure S2). This device operated in the spectral range of 350 to 2500 nm with a final spectral interval of 1 nm and spectral resolution of 3 nm below 1000 nm and 10 nm between 1000 and 2500 nm. To ensure accurate spectral measurements unaffected by atmospheric conditions, the device was calibrated using a 40 × 40 cm white calibration reference panel coated with white paint and barium sulfate (Labsphere, Inc., North Sutton, NH, USA) before conducting measurements. The device’s fiber optic probe with a 25° field of view (FOV) was placed vertically at a height of 80 cm above the canopy of each RIL/cultivar in a nadir orientation. This setup enabled the detection of spectral reflectance with a spot size of approximately 44 cm in diameter. The final reflectance curve for each RIL/cultivar in each replicate was measured from the central area of the second and third plant rows at three random places. Each measurement was an average of 10 scans. The average spectral reflectance of three sequential measurements and 10 scans per RIL/cultivar in each replicate in the range of 400–2500 nm was used to estimate seven vegetation SRIs and seven water SRIs, as shown in Table 6.

### 4.4. Genotypic Monitoring

#### 4.4.1. SSR Markers and Genomic DNA Extraction

In this study, we assessed the genetic diversity of 24 wheat germplasms using 60 SSR markers. These markers cover a majority of the chromosomes in hexaploid wheat genomes and were selected based on their association with salt tolerance in wheat, as reported in previous studies [40,88]. The sequences of these SSR markers are available on the Grain Genes website (http://wheat.pw.usda.gov; accessed on 11 January 2022) and are listed in Table S1. The genomic DNA of leaf samples was extracted from twenty-day-old seedlings using the Wizard Genomic DNA Purification Kit (PROMEGA Corporation Biotechnology, Madison, WI, USA). Following isolation, the samples were treated with RNase and stored at −20 °C. The concentration of the isolated DNA was determined at 260 nm using a UV/vis spectrophotometer (UV-2550, Shimadzu, Tokyo, Japan) and its quality was assessed by running it on a 0.8% agarose gel. The DNA stock was then adjusted to a final concentration of 25 ng µL^−1^.

**Table 6:** Description and references (ref.) of various vegetation and water spectral reflectance indices (SRIs) used in this study and derived from hyperspectral data.

Name and Abbreviation of SRIs	Formula	Ref.
Vegetation SRIs		
Blue normalized difference vegetation index (BNDVI)	(R_1245_ − R_415_)/(R_1245_ + R_415_)	[89]
Green normalized difference vegetation index (GNDVI)	(R_1245_ − R_550_)/(R_1245_ + R_550_)	[89]
Red normalized difference vegetation index (RNDVI)	(R_1245_ − R_680_)/(R_1245_ + R_680_)	[89]
Red-edge chlorophyll index (Chl_red-edge_)	(R_760_/R_710_) − 1	[1]
Enhanced vegetation index (EVI)	2.5 [(R_782_ − R_675_)/(R_782_ + 6 × R_675_ − 7.5 × R_445_ + 1)]	[90]
Modified transformed vegetation index (MTVI)	1.2 × [(1.2 × (R_800_ − R_550_) − 2.5 × (R_670_ − R_550_)_]_	[74]
Optimized soil adjusted vegetation index (OSAVI)	1.16 × (R_800_ − R_670_)/(R_800_ + R_670_ + 0.16)	[91]
Water SRIs		
Ratio water index (RWI)	(R_970_/R_900_)	[89]
Normalized water index (NWI)	(R_970_ − R_850_)/(R_970_ + R_850_)	[92]
Normalized difference water index (NDWI)	(R_737_ − R_1360_)/(R_737_ + R_1360_)	[89]
Normalized difference moisture index (NDMI)	(R_2200_ − R_1100_)/(R_2200_ + R_1100_)	[93]
Dry matter content index (DMCI)	(R_2305_ − R_1495_)/(R_2305_ + R_1495_)	[94]
Normalized multi-band drought index (NMDI)	860 − (R_1640_ − R_2130_)/860 + (R_1640_ − R_2130_)	[95]
Salinity and water stress index (SWSI)	(R_803_ − R_681_)/√ (R_1326_ − R_1507_)	[73]

#### 4.4.2. Polymerase Chain Reaction (PCR) Amplification and Gel Electrophoresis

The PCR was performed in a 20 µL reaction mixture containing 1.5 µL of DNA sample, 8 µL of nuclease-free water, 0.5 µL of both forward and reverse primers, and 10 µL of Green Master (Promega Corporation, Madison, WI, USA). The PCR protocol started with an initial denaturation at 94 °C for 4 min. This was followed by 40 cycles of denaturation at 94 °C for 1 min, annealing at the primer-specific temperature (as listed in Table S1 for each SSR primer) for 1 min, an extension at 72 °C for 2 min, and a final extension at 72 °C for 10 min before cooling to 4 °C. The products of PCR amplification were analyzed using capillary electrophoresis with the QI Axcel Advanced System Device (Qiagen, Hilden, Germany). The SSR markers’ amplified bands at each locus were visually scored for presence or absence with each primer. The scores were recorded in a binary matrix, with ‘1’ indicating a presence and ‘0’ indicating an absence of bands in each genotype.

### 4.5. Data Analysis

Prior to analysis, data distribution normality and variance homogeneity were assessed using the Kolmogorov–Smirnov test. Subsequently, a one-way analysis of variance (ANOVA) with a randomized complete block design followed by Tukey’s HSD post hoc test was conducted to determine significant differences in morpho-physiological traits and various SRIs among genotypes. Statistical analyses were conducted using SAS software (Version 9.2; Cary, NC, USA) with a significance threshold set to *p* < 0.05. The mean values of traits and SRIs for two seasons were plotted with standard error bars using Sigma Plot for Windows (version 11.0; Sysat Software Inc., Point Richmond, Chicago, IL, USA). The relationship between each SRI and the morpho-physiological traits was analyzed using linear and non-linear curve fitting. The equation with the highest R^2^ value was chosen as the best fit for this relationship. To enhance the clarity of the analysis as well as the relationships between morpho-physiological traits and different SRIs, we conducted a principal component analysis (PCA) on the mean values of morpho-physiological traits and SRIs across two seasons for all genotypes. This approach helps us understand the relationships between the traits and SRIs by grouping their contributions into major components rather than analyzing each SRI individually. The PCA was based on the correlation matrix data to reduce the dimensionality of the data space. We focused on the first two components of the PCA to capture the most variability in the data. Furthermore, two heatmap cluster analyses were conducted, one based on destructive morpho-physiological traits and the other on different SRIs. This grouping categorized the salt tolerance of tested genotypes using both destructive and time-consuming measurements (morpho-physiological traits), as well as quick and non-invasive assessments (SRIs).

PLSR models were used to evaluate four morpho-physiological traits. The models were created using the unscramble X software version 10.2 (CAMO-Software AS, Oslo, Norway). The input variables for the PLSR models were all SRIs, while the output variables were PDW, Chlt, LRWC, and GY. PLSR was conducted in conjunction with LOOCV to establish the relationship between the inputs and outputs. Determining the optimal number of latent factors (ONLFs) is crucial in PLSR analysis to accurately represent the calibration data without overfitting or underfitting. In this study, the ONLFs for each morpho-physiological variable in the PLSR analysis were identified using leave-one-out cross-validation (LOOCV). The optimal ONLFs were determined by minimizing the predicted residual error sum of squares to achieve the lowest value. To enhance the reliability of the findings, a random 10-fold cross-validation was performed on the data collected over two seasons. In the model training process, 25% of the data were allocated for validation (val.) and 75% for calibration (cal.). The model with the lowest root mean squared error (RMSE) and the highest coefficient of determination (R^2^) was chosen as the best fit for both cal. and val. datasets.

A dissimilarity matrix was generated using the Jaccard dissimilarity coefficient to assess the pairwise genetic dissimilarity among genotypes. Subsequently, agglomerative hierarchical clustering was conducted using the unweighted pair group average method (UPGMA) within the same statistical package. A Mantel test was conducted to compare the genetic distance matrices derived from SSR data with measurements of morpho-physiological traits and SRIs. The SSR markers closely linked to each morpho-physiological trait or SRI were identified using a stepwise multiple linear regression analysis. The morpho-physiological traits or SRIs were considered dependent variables, while the molecular marker observations were treated as independent variables. The significance of the coefficients of determination (R^2^) was assessed at a 5% probability level.

The genotypic (GCV) and phenotypic coefficients of variability (PCV) were calculated for each morpho-physiological trait and SRI. Traits and SRIs with values greater than 20% were considered high, those with values less than 10% were considered low, and values between 10% and 20% were considered medium. The high values of the GCV and PCV for the traits and SRIs indicate that these traits can be improved through selection. It is important to note that when the PCV value of a trait and SRI exceeds the corresponding GCV, it suggests that environmental factors play a significant role in the expression of that trait and SRI. The GCV and PCV were estimated using the formula of Singh et al. [96].

(3) $$GCV=\frac{\sqrt{\sigma^{2}g}}{\overset{–}{X}}\times 100$$

(4) $$PCV=\frac{\sqrt{\sigma^{2}p}}{\overset{–}{X}}\times 100$$

Genotypic (σ^2^g) and phenotypic (σ^2^p) variances were calculated using the formulas provided by Fehr [97]. σ^2^g was determined by subtracting the mean squared error (EMS) from the mean squared values of genotype (GMS) and dividing it by the number of replicates. σ^2^p was calculated as the sum of σ^2^g and EMS.

The broad-sense heritability (h^2^) was calculated across two seasons using the formula developed by Fehr [97] to determine the proportion of phenotypic variance due to genetic effects.

(5) $$h^{2}=(\sigma^{2}g)/(\sigma^{2}g+\frac{\sigma^{2}gy}{y}+\frac{\sigma^{2}e}{yr})$$

The genetic gain (GG) was calculated using the following equation:

(6) $$GG=\frac{GA}{\overset{–}{X}}\times 100$$

where k is the selection differential (2.06 at 5% selection intensity) and $\overset{–}{X}$ is the phenotypic mean for morpho-physiological traits and SRIs.

## 5. Conclusions

This study suggests that SRIs can serve as effective high-throughput phenotyping tools for quickly assessing the salt tolerance of multiple wheat genotypes in real field conditions. SRIs offer a fast and cost-effective alternative to the traditional, labor-intensive methods of measuring morpho-physiological traits that often require destructive sampling. The results led to the following conclusions:

1. The different SRIs showed significant differences among the tested RILs/cultivars, as did the morpho-physiological traits.
2. Genetic analysis of morpho-physiological traits has proven to be an effective screening criterion for distinguishing salt tolerance among RILs/cultivars in real field conditions.
3. The genetic basis of various SRIs in wheat suggests that incorporating them into breeding programs could result in rapid genetic improvements.
4. The different SRIs were able to effectively group wheat RILs/cultivars based on their salt tolerance, similar to the morpho-physiological traits.
5. High-throughput and non-destructive SRIs can assess wheat genotypes’ salinity tolerance by the indirect assessment of morpho-physiological traits.
6. Genetic analysis and SSR markers have shown that SRIs can be effectively integrated into wheat breeding programs as valuable tools for indirectly selecting and improving salinity tolerance in wheat crops.
7. The combination of PLSR models with all SRIs showed the highest performance in estimating various morpho-physiological traits in both the calibration and validation datasets.
8. While the results of this study regarding the ability of SRIs for the indirect assessment of morpho-physiological traits may not be universally applicable to all combinations of salinity levels and crop genotypes, they present an intriguing opportunity to enhance cultivar selection for specific site conditions and genotype panels. This approach could potentially reduce the need for the labor-intensive, expensive, and time-consuming destructive calibration of morpho-physiological traits. By analyzing a smaller subset of data destructively, it may be possible to predict parameters such as PDW, LRWC, Chlt, and GY for a larger set of cultivars, leading to significant cost and time savings essential for high-throughput phenotyping in breeding selection processes. Further research to explore the implications of these findings would be highly beneficial.

## Figures and Tables

**Table 1:** Mean square values for the effects of the season (S), replications (R), genotypes (G), and their possible interactions using ANOVA on four destructively measured traits, seven vegetation SRIs, and seven water SRIs of 24 wheat RILs/cultivars evaluated for each growing season and the combined analysis of two seasons under salinity conditions.

Traits	First Season			Second Season			Combined Two Seasons				
	R	G	Error	R	G	Error	S	R (S)	G	S × G	Error
Df	2	23	46	2	23	46	1	4	23	23	92
Morpho-physiological traits											
**PDW**	0.2070	1.1361 ***	0.0873	0.0471	0.8132 ***	0.1052	0.0513 ^ns^	0.1271	1.9068 ***	0.0426 ^ns^	0.0963
**LRWC**	4.6177	58.7985 ***	7.9292	1.7866	44.6748 ***	3.5207	69.5417 ***	3.2021	82.7451 ***	20.7282 ***	5.725
**Chlt**	0.0588	0.9458 ***	0.0267	0.2431	0.9814 ***	0.0856	1.8405 ***	0.1509	1.6196 ***	0.3076 ***	0.0561
**GY**	0.0219	0.6805 ***	0.0425	0.0974	0.8121 ***	0.0547	0.1002 ^ns^	0.0596	1.4730 ***	0.0196 ^ns^	0.0486
Vegetation spectral reflectance indices (vegetation SRIs)											
**BNDVI**	0.0005	0.0130 ***	0.0011	0.0004	0.0147 ***	0.0013	0.0072 *	0.0004	0.0243 ***	0.0034 ***	0.0012
**GNDVI**	0.0003	0.0244 ***	0.0004	0.0005	0.0209 ***	0.0016	0.0087 **	0.0004	0.0403 ***	0.0049 ***	0.001
**RNDVI**	0.0009	0.0369 ***	0.0017	0.0003	0.0157 ***	0.0013	0.0802 ***	0.0006	0.0468 ***	0.0058 ***	0.0015
**Chl_red-edge_**	0.0205	1.3536 ***	0.1077	0.3546	1.2817 ***	0.1736	24.5025 ***	0.1876	2.4248 ***	0.2105 *	0.1407
**EVI**	0.0030	0.0552 ***	0.0027	0.0009	0.0162 ***	0.0014	0.3249 ***	0.0019	0.0609 ***	0.0106 ***	0.0021
**MTVI**	0.0011	0.0575 ***	0.0032	0.0046	0.0313 ***	0.0016	0.2320 ***	0.0028	0.0785 ***	0.0104 ***	0.0024
**OSAVI**	0.0003	0.0424 ***	0.0016	0.0012	0.0186 ***	0.0014	0.1885 ***	0.0007	0.0524 ***	0.0086 ***	0.0015
Water spectral reflectance indices (water SRIs)											
**WI**	0.1170	1.0162 ***	0.0831	0.0901	1.8791 ***	0.2592	14.6944 ***	0.1035	2.5100 ***	0.3852 **	0.1711
**NWI**	0.0001	0.0003 ***	0.0001	0.0001	0.0003 ***	0.0001	0.0032 ***	0.0001	0.0005 ***	0.0001 **	0.0001
**NDWI**	0.0047	0.0227 ***	0.0016	0.0002	0.0477 ***	0.0027	0.6493 ***	0.0024	0.0624 ***	0.0080 ***	0.0021
**NDMI**	0.0022	0.0217 ***	0.0020	0.0021	0.0260 ***	0.0011	0.2216 ***	0.0022	0.0449 ***	0.0028 *	0.0016
**DMCI**	0.0011	0.0158 ***	0.0022	0.0016	0.0032 **	0.0011	0.0002 ^ns^	0.0014	0.0144 ***	0.0046 ***	0.0017
**NMDI**	0.0009	0.0159 ***	0.0011	0.0021	0.0167 ***	0.0014	0.0779 ***	0.0015	0.0302 ***	0.0024 *	0.0013
**SWSI**	0.0328	0.1364 ***	0.0095	0.0038	0.1501 ***	0.0216	0.8235 ***	0.0183	0.2555 ***	0.0311 *	0.0156

PDW, LRWC, Chlt, and GY indicate plant dry weight (g plant^−1^), leaf relative water content (%), total chlorophyll content (mg g^−1^ FW), and grain yield (ton ha^−1^), respectively. The full names of the abbreviations of the SRIs are listed in Table 6 in the Materials and Methods Section. *, **, *** indicates significance at the 0.05, 0.01, and 0.001 probability levels, respectively, and ns indicates not significant.

**Table 2:** Broad-sense heritability (h^2^), genotypic coefficient of variance (GCV), phenotypic coefficient of variance (PCV), and genetic gain (GG) for morpho-physiological traits and different spectral reflectance indices (SRIs) across two seasons.

Traits/SRIs	h^2^	GCV	PCV	GG
Morpho-physiological traits				
PDW	77.57	4.29	4.87	7.78
LRWC	48.51	1.35	1.94	1.94
Chlt	61.84	7.26	9.23	11.76
GY	84.01	5.46	5.96	10.32
Vegetation SRIs				
BNDVI	60.93	2.54	3.28	4.05
GNDVI	77.71	3.72	4.22	6.75
RNDVI	78.69	3.84	4.33	7.01
Chl_red-edge_	57.16	7.80	10.31	12.14
EVI	66.30	4.32	5.31	7.25
MTVI	76.83	5.60	6.39	10.12
OSAVI	67.49	4.04	4.92	6.84
Water SRIs				
RWI	51.05	7.76	10.86	11.42
NWI	75.35	−5.41	−6.23	−9.67
NDWI	78.25	−5.12	−5.78	−9.32
NDMI	70.32	−4.90	−5.85	−8.47
DMCI	21.34	−4.24	−9.18	−4.04
NMDI	65.53	4.11	5.12	6.81
SWSI	69.21	7.28	8.75	12.48

PDW, LRWC, Chlt, and GY indicate plant dry weight (g plant^−1^), leaf relative water content (%), total chlorophyll content (mg g^−1^ FW), and grain yield (ton ha^−1^), respectively. The full names of the abbreviations for the different SRIs are listed in Table 6 in the Materials and Methods Section.

**Table 3:** Association of the most influential SSR markers with destructively measured morpho-physiological traits and different spectral reflectance indices (SRIs).

Morpho-Physiological Traits				Vegetation SRIs				Water SRIs			
Traits	Markers	R^2^ Par	R^2^ Cum	Traits	Markers	R^2^ Par	R^2^ Cum	Traits	Markers	R^2^ Par	R^2^ Cum
PDW	Gwm55	0.26 ***	0.75	BNDVI	Gwm350	0.30 ***	0.71	WI	Barc44	0.51 ***	0.86
	Wmc154	0.18 ***			Wmc245	0.29 *			Cfd9	0.21 **	
	Wmc405	0.12 *			Wmc154	0.11 ***			Barc167	0.09 ***	
	Barc110	0.12 **		GNDVI	Barc44	0.49 ***	0.76		Cfd49	0.06 *	
	Cfd18	0.07 *			Cfd9	0.19 **		NDWI	Barc44	0.42 ***	0.89
LRWC	Barc44	0.36 ***	0.62		Barc167	0.08 *			Cfd9	0.30 ***	
	Wmc11	0.18 *		RNDVI	Gwm335	0.41 ***	0.86		Wmc405	0.07 ***	
	Cfd9	0.09 *			Cfd9	0.18 **			Wmc154	0.04 ***	
Chlt	Wmc245	0.24 *	0.39		Barc167	0.14 ***			Gwm539	0.05 ***	
	Cfd9	0.15 ***			Gwm210	0.06 ***		NWI	Barc44	0.40 ***	0.56
GY	Barc44	0.37 ***	0.66		Wmc18	0.06 *			Cfd9	0.16 *	
	Cfd9	0.15 *		Chl_red-edge_	Gwm335	0.37 ***	0.84	DMCI	Barc44	0.37 ***	0.67
	Barc34	0.14 *			Cfd9	0.24 ***			Barc34	0.16 **	
					Cfd66	0.08 ***			Cfd9	0.14 *	
					Wmc154	0.06 ***		NMDI	Gwm350	0.33 ***	0.86
					Wmc405	0.04 **			Wmc154	0.21 ***	
					Gwm55	0.03 **			Cfd18	0.15 **	
					Gwm314	0.02 *			Gwm314	0.06 *	
				EVI	Gwm335	0.39 ***	0.65		Cfd9	0.07 ***	
					Cfd9	0.27 ***			Wmc11	0.05 *	
				MTVI	Barc44	0.42 ***	0.79	SWSI	Barc44	0.42 ***	0.74
					Cfd9	0.23 **			Cfd9	0.27 **	
					Barc167	0.09 *			Wmc405	0.06 *	
					Gwm296	0.05 *		NDMI	Barc44	0.43 *	0.97
				OSAVI	Cfd9	0.56 ***	0.8		Wmc405	0.19 *	
					Gwm210	0.10 ***			Wmc154	0.11 *	
					Wmc154	0.08 ***			Barc167	0.10 *	
					Wmc503	0.06 ***			Gwm539	0.08 *	
									Gwm314	0.06 *	

PDW, LRWC, Chlt, and GY indicate plant dry weight (g plant^−1^), leaf relative water content (%), total chlorophyll content (mg g^−1^ FW), and grain yield (ton ha^−1^), respectively. The full names of the abbreviations for the different SRIs are listed in Table 6 in the Materials and Methods Section. *, **, *** indicates significance at the 0.05, 0.01, and 0.001 probability levels, respectively.

**Table 4:** The best formulae and determination coefficients (R^2^) for relationships between different spectral reflectance indices (SRIs) and each morpho-physiological trait.

Vegetation SRIs				Water SRIs			
SRIs	Traits	Formulae	R^2^	SRIs	Traits	Formulae	R^2^
BNDVI	PDW	y = 6.48x − 0.03	0.54 ***	RWI	PDW	y = −0.38x^2^ + 2.59x + 1.32	0.40 **
	Chlt	y = 5.16x − 2.29	0.40 **		Chlt	y = 0.39x + 0.99	0.22 *
	LRWC	y = 236.95x^2^ − 359.44x + 203	0.42 **		LRWC	y = 3.48x + 59.72	0.36 **
	GY	y = 6.96x − 2.14	0.80 ***		GY	y = 0.66x + 1.93	0.72 ***
GNDVI	PDW	y = 4.88x + 1.83	0.50 ***	NWI	PDW	y = −42.7x + 3.05	0.50 ***
	Chlt	y = 25.68x^2^ − 33.64x + 12.72	0.48 **		Chlt	y = −28.23x + 0.48	0.25 *
	LRWC	y = 145.51x^2^ − 186.48x + 126.68	0.40 **		LRWC	y = −253.32x + 55.14	0.40 **
	GY	y = 5.28x − 0.18	0.76 ***		GY	y = −48.69x + 1.01	0.84 ***
RNDVI	PDW	y = 3.90x + 2.43	0.37 **	NDWI	PDW	y = 3.79x + 3.72	0.47 ***
	Chlt	y = 28.81x^2^ − 40.74x + 16.11	0.47 **		Chlt	y = 2.33x + 0.99	0.21 *
	LRWC	y = 210.55x^2^ − 305.03x + 177.68	0.31 *		LRWC	y = 20x + 60.24	0.30 **
	GY	y = 4.74x + 0.06	0.71 ***		GY	y = 4.25x + 1.79	0.77 ***
Chl_rededge_	PDW	y = 0.66x + 3.44	0.54 ***	NDMI	PDW	y = −4.49x + 2.42	0.47 ***
	Chlt	y = 0.28x^2^ − 1.09x + 2.77	0.53 ***		Chlt	y = −2.67x + 0.26	0.19 *
	LRWC	y = 3.18x + 59.66	0.32 **		LRWC	y = 155.2x^2^ + 177.4x + 117.32	0.40 **
	GY	y = 0.67x + 1.71	0.72 ***		GY	y = −4.78x + 0.52	0.69 ***
EVI	PDW	y = 3.79x + 2.79	0.46 ***	DMCI	PDW	y = −119.16x^2^ − 68.81x − 4.03	0.56 **
	Chlt	y = 14.87x^2^ − 16.98x + 6.51	0.53 ***		Chlt	y = −4.93x + 0.84	0.21 *
	LRWC	y = 56.67x^2^ + 61.25x + 83.9	0.23 *		LRWC	y = −50.45x + 56.79	0.44 ***
	GY	y = 4.10x + 0.87	0.69 ***		GY	y = −9.61x + 1.34	0.90 ***
MTVI	PDW	y = 3.46x + 3.11	0.50 ***	NMDI	PDW	y = 5.38x + 1.98	0.46 ***
	Chlt	y = 18.89x^2^ − 21.70x + 7.86	0.68 ***		Chlt	y = 3.89x − 0.45	0.28 **
	LRWC	y = 66.93x^2^ − 69.45x + 84.86	0.37 **		LRWC	y = 30.16x + 49.93	0.33 **
	GY	y = 3.74x + 1.21	0.74 ***		GY	y = 6.41x − 0.39	0.84 ***
OSAVI	PDW	y = 4.68x + 2.15	0.60 ***	SWSI	PDW	y = 1.71x + 2.69	0.38 **
	Chlt	y = 25.35x^2^ − 31.51x + 11.46	0.62 ***		Chlt	y = 1.12x + 0.25	0.19 *
	LRWC	y = 108.35x^2^ − 128.7x + 105.3	0.36 **		LRWC	y = 25.1x^2^ − 69.44x + 114.91	0.38 **
	GY	y = 4.69x + 0.43	0.78 ***		GY	y = 2.17x + 0.23	0.81 ***

PDW, LRWC, Chlt, and GY indicate plant dry weight (g plant^−1^), leaf relative water content (%), total chlorophyll content (mg g^−1^ FW), and grain yield (ton ha^−1^), respectively. The full names of the abbreviations for the different SRIs are listed in Table 6 in the Materials and Methods Section. *,**, *** indicate significance at the 0.05, 0.01, and 0.001 probability levels, respectively.

**Table 5:** Calibration and cross-validation statistics of partial least squares regression (PLSR) models based on all spectral reflectance indices (SRIs) for estimating different morpho-physiological traits.

Traits	Optimal SRIs	Parameters	Training		Cross Validation	
			R^2^	RMSE	R^2^	RMSE
PDW	RNDVI, OSAVI	(2, 8, relu)	0.72 ***	0.291	0.66 **	0.323
Chlt	MTVI	(5, 10, relu)	0.81 ***	0.224	0.75 ***	0.241
LRWC	BNDVI	(3, 12, tanh)	0.85 ***	1.417	0.68 ***	2.032
GY	OSAVI, NMDI, DMCI	(3, 10, relu)	0.96 ***	0.103	0.94 ***	0.111

PDW, LRWC, Chlt, and GY indicate plant dry weight (g plant^−1^), leaf relative water content (%), total chlorophyll content (mg g^−1^ FW), and grain yield (ton ha^−1^), respectively. The full names of the abbreviations for the different SRIs are listed in Table 6 in the Materials and Methods Section. ** and *** indicate significance at the 0.01 and 0.001 probability levels, respectively.

## Data Availability

All data are presented within the article.

## Supplementary Materials

The following supporting information can be downloaded at: https://www.mdpi.com/article/10.3390/plants13182610/s1, Figure S1: Average daily climatic data from December to April from the experimental field during wheat’s growth stages over two growing seasons. DAS indicates days after sowing; Figure S2: (A) shows an overview of the experimental field; (B) shows canopy spectral reflectance measurements; Table S1: List of SSR markers used in this study across 24 wheat genotypes, including marker sequence and annealing temperature.
