# Supplementary material for: Integrating Hyperspectral Reflectance-Based Phenotyping and SSR Marker-Based Genotyping for Assessing the Salt Tolerance of Wheat Genotypes under Real Field Conditions
Source: Plants (Basel). 2024 Sep 19;13(18):2610. doi: 10.3390/plants13182610 (PMC11435290; doi:10.3390/plants13182610)
Supplement: Supplementary file 1 [file plants-13-02610-s001.zip › plants-3153004-supplementary.pdf]

Table S1: List of SSR markers used in this study across 24 wheat genotypes, including marker sequence and annealing temperature.

Oligo	Chromosome	Sequence 5 to 3		Annealing	Repeat
		F	R		
cslinkkna2	5A	TCTCCATCATTCAACATCAATCG	TGTAGCTCGTCGGGGTGTGTTGC	58	-
Xbarc182	7B	CCATGGCCAACAGCTCAAGGTCTC	CGCAAAACCGCATCAGGGAAGCACCAAT	58	(CT)15
Xcfd1	6B,6D,6A	ACCAAAGAACTTGCCTGGTG	AAGCCTGACCTAGCCCAAAT	60	(GCC)6
Xcfd13	6B,6D	CCACTAACCAAGCTGCCATT	TTTTTGGCATTGATCTGCTG	60	(CT)20(TGTA)3
Xcfd18	5D	CATCCAACAGCACCAAGAGA	GCTACTACTATTTCATTGCGACCA	60	(GA)25
Xcfd183	5D	ACTTGCACTTGCTATACTTACGAA	GTGTGTCGGTGTGTGGAAAG	60	(CA)22
Xcfd19	1,5,6D	TACGCAGGTTTGCTGCTTCT	GGAGTTCACAAGCATGGGTT	60	(GA)18
Xcfd46	7D	TGGTGGTATAGTCGTTGGAGC	CCACACACACACACCATCAA	60	(GT)29
Xcfd49	6D	TGAGTTCTTCTGGTGAGGCA	GAATCGGTTCACAAGGGAAA	60	(GA)33
Xcfd60	6D,5B	TGACCGGCATTCAGTATCAA	TGGTCACTTTGATGAGCAGG	60	(CA)25
Xcfd66	7D	AGGTCTTGGTGGTTTTGGTG	TTTTCACATGCCCACAGTTG	60	(GC)9(AG)60
Xcfd9	3D	TTGCACGCACCTAAACTCTG	CAAGTGTGAGCGTCGG	60	(TC)29
Xgwm133	3A,6,7B,6D	ATCTAAACAAGACGGCGGTG	ATCTGTGACAACCGGTGAGA	60	(CT)39imp
Xgwm148	2B	GTGAGGCAGCAAGAGAGAAA	CAAAGCTTGACTCAGACCAAA	60	(CA)22
Xgwm174	5D	GGGTTCCTATCTGGTAAATCCC	GACACACATGTTCCTGCCAC	55	(CT)22
Xgwm181	3B	TCA TTG GTA ATG AGG AGA GA	GAA CCA TTC ATG TGC ATG TC	51	(GA)28
Xgwm205	5D,5A	CGACCCGGTTCACTTCAG	AGTCGCCGTTGTATAGTGCC	60	(CT)21
Xgwm210	2A	TGCATCAAGAATAGTGTGGAAG	TGAGAGGAAGGCTCACACCT	55	(GA)20
Xgwm247	3A,3B	GCAATCTTTTTTCTGACCACG	ATG TGC ATG TCG GAC GC	55	(GA)24
Xgwm249	2D	CAAATGGATCGAGAAAGGGA	CTGCCATTTTTCTGGATCTACC	55	(GA)11(GGA)8
Xgwm296	2D,7D	AATTCAACCTACCAATCTCTG	GCCTAATAAACTGAAAACGAG	55	(CT)28
Xgwm299	3B,2B	ACTACTTAGGCCTCCCGCC	TGACCCACTTGCAATTCATC	55	(GA)31(TAG)4
Xgwm312	2A	ATCGCATGATGCACGTAGAG	ACATGCATGCCTACCTAATGG	60	(GA)37
Xgwm314	3D,4B	AGGAGCTCCTCTGTGCCAC	TTCGGGACTCTCTTCCCTG	55	(CT)25imp
Xgwm335	5B	CGTACTCCACTCCACACGG	CGGTCCAAGTGCTACCTTTC	55	(GA)14(GCGT)3
Xgwm340	3B	GCAATCTTTTTCTGACCACG	ACGAGGCAAGAACACACATG	60	(GA)26

Xgwm350	7D	ACCTCATCCACATGTTCTACG	GCATGGATAGGACGCCC	55	(GT)14
Xgwm413	1B,1A	TGCTTGTCTAGATTGCTTGGG	GATCGTCTCGTCCTTGGCA	60	(GA)18
Xgwm455	2D,6B	ATTCGGTTCGCTAGCTACCA	ACGGAGAGCAACCTGCC	55	(GT)19imp
Xgwm539	2D	CTGCTCTAAGATTCATGCAACC	GAGGCTTGTGCCCTCTGTAG	60	(GA)27
Xgwm614	2B,2A,2D	GATCACATGCATGCGTCATG	TTTTACCGTTCCGGCCTT	60	(GA)23imp
Xgwm674	3A	TCGAGCGATTTTTCCTGC	TGACCGAGTTGACCAAAACA	60	(CT)16CCC(GT)4
Xwmc11	3A, 3D	TTGTGATCCTGGTTGTGTTGTGA	CACCCAGCCGTTATATATGTTGA	61	-
Xwmc169	3, 5A	TACCCGAATCTGGAAAATCAAT	TGGAAGCTTGCTAACTTTGGAG	61	(CA)25 65 to 114
Xwmc17	7A, 7B	ACCTGCAAGAAATTAGGAACTC	CTAGTGTTTCAAATATGTCGGA	51	-
Xwmc170	2A,2D	ACATCCACGTTTATGTTGTTGC	TTGGTTGCTCAACGTTTACTTC	61	(CA)19 378 to 415
Xwmc18	2D	CTGGGGCTTGGATCACGTCATT	AGCCATGGACATGGTGTCCTTC	61	(CA)(CT) CM*
Xwmc367	1B	CTGACGTTGATGGGCCACTATT	GTGGTGGAAGAGGAAGGAGAGG	61	(GCC)5 125 to 139
Xwmc419	4B	GTTTCGGATAAAACCGGAGTGC	ACTACTTGTGGGTTATCACCAGCC	61	(GA)16 111 to 142
Xwmc432	1D	ATGACACCAGATCTAGCAC	AATATTGGCATGATTACACA	51	(GT)14 238 to 265
Xwmc44	1B	GGTCTTCTGGGCTTTGATCCTG	TGTTGCTAGGGACCCGTAGTGG	61	(GT)35 341 to 410
Xwmc503	2D	GCAATAGTTCCCGCAAGAAAAG	ATCAACTACCTCCAGATCCCGT	61	(GT)11 112 to 133
Xwmc661	2B	CCACCATGGTGCTAATAGTGTC	AGCTCGTAACGTAATGCAACTG	61	-

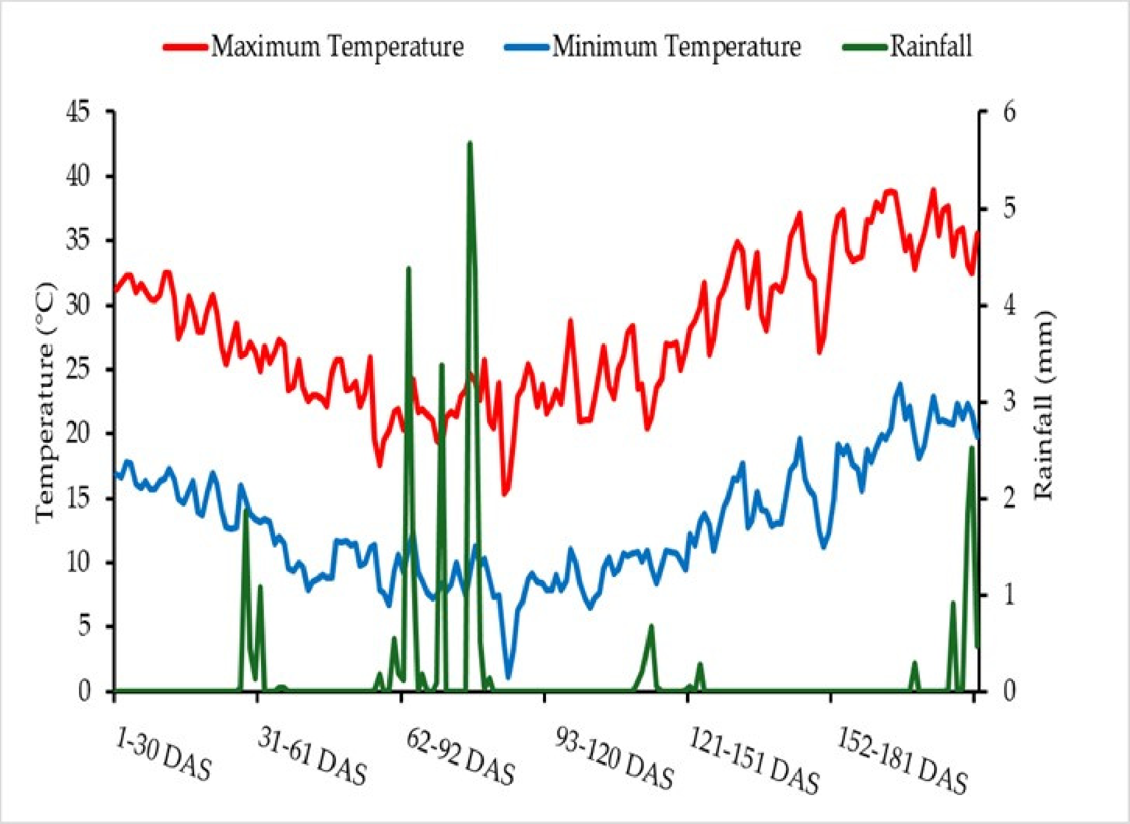

Figure S1. Average daily climatic data from December to April at the Experimental field during wheat's growth stages over two growing seasons. DAS indicates days after sowing.

.

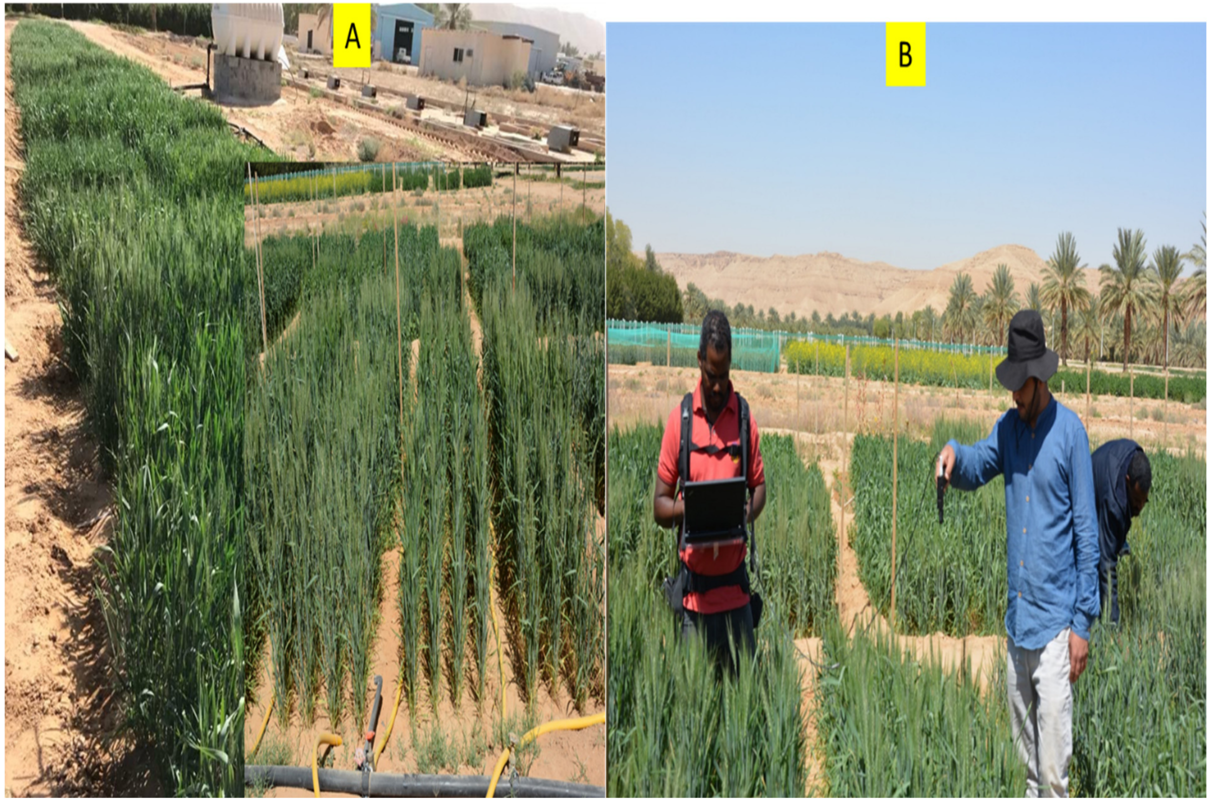

Figure S2. (A) shows an overview of the experimental field; (B) shows canopy spectral reflectance measurements.
